# GWAS Identifies Risk Locus for Erectile Dysfunction and Implicates Hypothalamic Neurobiology and Diabetes in Etiology

**DOI:** 10.1016/j.ajhg.2018.11.004

**Published:** 2018-12-21

**Authors:** Jonas Bovijn, Leigh Jackson, Jenny Censin, Chia-Yen Chen, Triin Laisk, Samantha Laber, Teresa Ferreira, Sara L. Pulit, Craig A. Glastonbury, Jordan W. Smoller, Jamie W. Harrison, Katherine S. Ruth, Robin N. Beaumont, Samuel E. Jones, Jessica Tyrrell, Andrew R. Wood, Michael N. Weedon, Reedik Mägi, Benjamin Neale, Cecilia M. Lindgren, Anna Murray, Michael V. Holmes

**Affiliations:** 1 Big Data Institute at the Li Ka Shing Centre for Health Information and Discovery, University of Oxford, Oxford OX3 7LF, UK; 2 Wellcome Centre for Human Genetics, Nuffield Department of Medicine, University of Oxford, Oxford OX3 7BN, UK; 3 Institute of Biomedical and Clinical Science, University of Exeter Medical School, University of Exeter, Exeter EX2 5DW, UK; 4 Analytic and Translational Genetics Unit, Massachusetts General Hospital, Boston, MA 02114, USA; 5 Psychiatric & Neurodevelopmental Genetics Unit, Massachusetts General Hospital, Boston, MA 02114, USA; 6 Estonian Genome Center, Institute of Genomics, University of Tartu, Tartu 51010, Estonia; 7 Department of Obstetrics and Gynecology, Institute of Clinical Medicine, University of Tartu, Tartu 50406, Estonia; 8 Program in Medical and Population Genetics, Broad Institute, Cambridge, MA 02142, USA; 9 Department of Genetics, University Medical Center Utrecht, Utrecht, the Netherlands; 10 Genetics of Complex Traits, University of Exeter Medical School, University of Exeter, Exeter EX2 5DW, UK; 11 National Institute for Health Research Oxford Biomedical Research Centre, Oxford University Hospital, Old Road, Oxford OX3 7LE, UK; 12 Clinical Trial Service Unit & Epidemiological Studies Unit (CTSU), Nuffield Department of Population Health, Big Data Institute Building, Roosevelt Drive, University of Oxford, Oxford OX3 7LF, UK; 13 Medical Research Council Population Health Research Unit at the University of Oxford, Nuffield Department of Population Health, University of Oxford, Oxford, UK

**Keywords:** erectile dysfunction, impotence, diabetes, SIM1, GWAS, genome-wide association, Mendelian randomization, mendelian randomisation, UK biobank

## Abstract

Erectile dysfunction (ED) is a common condition affecting more than 20% of men over 60 years, yet little is known about its genetic architecture. We performed a genome-wide association study of ED in 6,175 case subjects among 223,805 European men and identified one locus at 6q16.3 (lead variant rs57989773, OR 1.20 per C-allele; p = 5.71 × 10^−14^), located between *MCHR2* and *SIM1*. *In silico* analysis suggests *SIM1* to confer ED risk through hypothalamic dysregulation. Mendelian randomization provides evidence that genetic risk of type 2 diabetes mellitus is a cause of ED (OR 1.11 per 1-log unit higher risk of type 2 diabetes). These findings provide insights into the biological underpinnings and the causes of ED and may help prioritize the development of future therapies for this common disorder.

## Main Text

Erectile dysfunction (ED) is the inability to develop or maintain a penile erection adequate for sexual intercourse.^1^ ED has an age-dependent prevalence, with 20%–40% of men aged 60–69 years affected.^1^ The genetic architecture of ED remains poorly understood, owing in part to a paucity of well-powered genetic association studies. Discovery of such genetic associations can be valuable for elucidating the etiology of ED and can provide genetic support for potential new therapies.

We conducted a genome-wide association study (GWAS) in the population-based UK Biobank (UKBB) and the Estonian Genome Center of the University of Tartu (EGCUT) cohorts and hospital-recruited Partners HealthCare Biobank (PHB) cohort. Subjects in UKBB were of self-reported white ethnicity, with subjects in EGCUT and PHB of European ancestry, as per principal components analyses (Supplemental Material and Methods).

ED was defined as self-reported or physician-reported ED using ICD10 codes N48.4 and F52.2, or use of oral ED medication (sildenafil/Viagra, tadalafil/Cialis, or vardenafil/Levitra), or a history of surgical intervention for ED (using OPCS-4 codes L97.1 and N32.6) (Supplemental Material and Methods). The prevalence of ED in the cohorts was 1.53% (3,050/199,352) in UKBB, 7.04% (1,182/16,787) in EGCUT, and 25.35% (1,943/7,666) in PHB (Table S1). Demographic characteristics of the subjects in each cohort are shown in Table S2. The reasons for the different prevalence rates in the three cohorts may include a higher median cohort age for men in PHB (65 years, compared to 59 years in UKBB and 42 years in EGCUT; Table S2), “healthy volunteer” selection bias in UKBB,^2^ a lack of primary care data availability in UKBB, and intercultural differences, including “social desirability” bias.^3^, ^4^ Importantly, we note that the assessment of exposure-outcome relationships remains valid, despite the prevalence likely not being representative of the general population prevalence.

**Figure 1:**
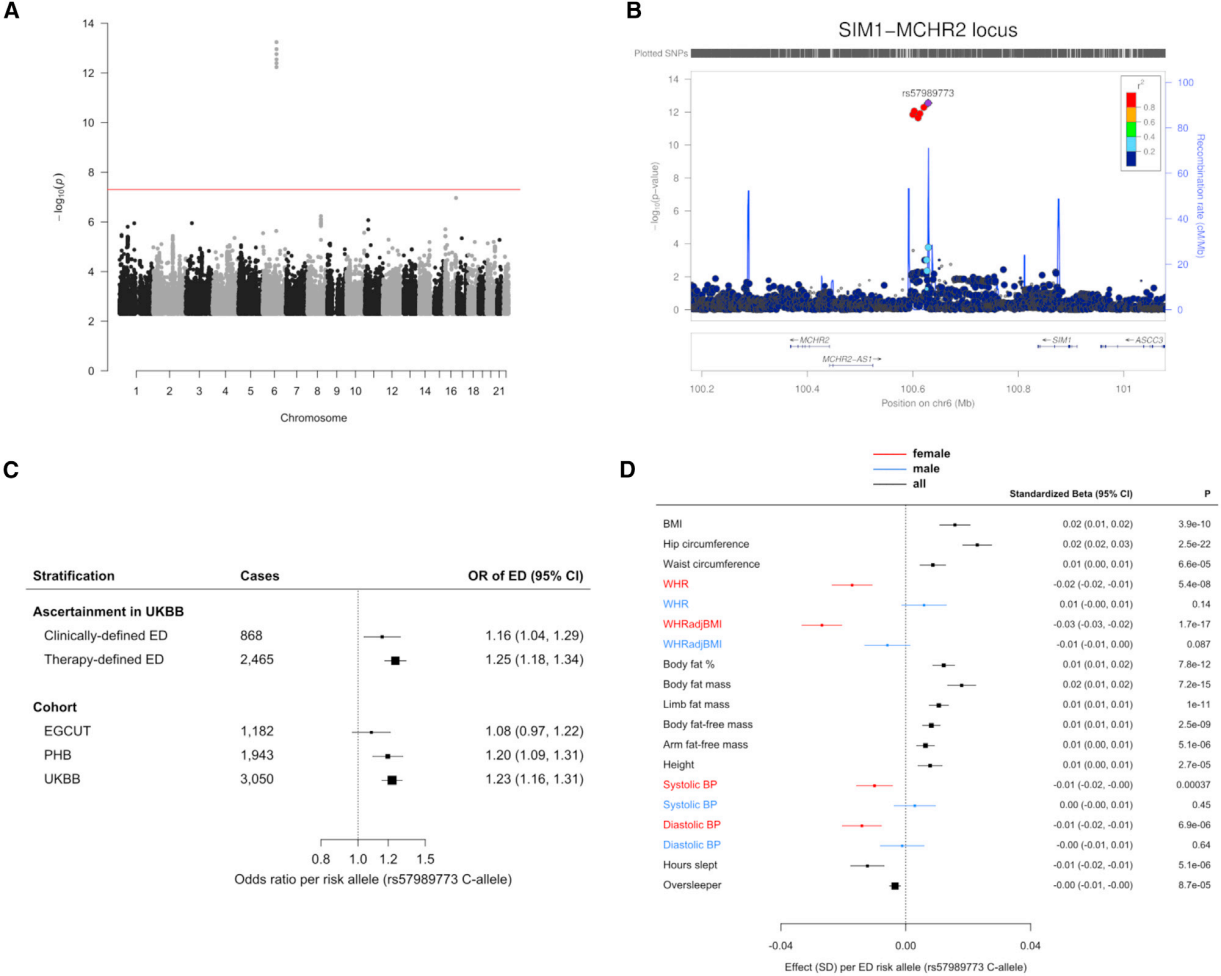
6q16.3 (Lead Variant rs57989773) Is an Erectile Dysfunction-Associated Locus and Exhibits Pleiotropic Phenotypic Effects (A) Genome-wide meta-analysis revealed a single genome-wide significant locus for ED at 6q16.3. Only variants with a p value for association of <0.005 are shown. The red line indicates the genome-wide association significance threshold (set at 5 × 10^−8^). (B) Six genome-wide significant variants at 6q16.3 are in high LD. (C) The association of rs57989773 with ED shows a consistent direction of effect across the three cohorts and across clinically and therapy defined ED in UKBB. Estimates are per C-allele. Boxes represent point estimates of effects. Box sizes are drawn proportional to the precision of the estimates. Lines represent 95% confidence intervals. (D) PheWAS reveals sex-specific associations of rs57989773 with waist-hip ratio and blood pressure. A PheWAS of 105 predefined traits using the lead ED SNP rs57989773 found associations with 12 phenotypes at p < 4.8 × 10^−4^ (surpassing the Bonferroni-corrected threshold of 0.05/105; Table S4). All allelic estimates are aligned to the ED risk allele (i.e., C-allele of rs57989773). Due to the nature of the ED phenotype and previously reported sex-specific effects in the *MCHR2-SIM1* locus,^5^ sex-specific analyses were performed in significant traits. Diastolic blood pressure (DBP) and systolic blood pressure (SBP) are included here (despite not meeting the Bonferroni-corrected threshold in the original analysis) due to previous reports of effects on blood pressure in individuals with rare, coding variants in *SIM1*. Sexual heterogeneity was found to be present (surpassing a Bonferroni-corrected threshold of 0.05/7 for the number of traits where sex-specific analyses were conducted) for DBP (p value_heterogeneity_ = 6.52 × 10^−3^), SBP (p value_heterogeneity_ = 3.73 × 10^−3^), waist to hip ratio (WHR; p value_heterogeneity_ = 2.39 × 10^−6^), and WHR adjusted for BMI (p value_heterogeneity_ = 1.77 × 10^−5^). This plot shows sex-specific estimates only for traits showing presence of sexual heterogeneity. Continuous traits were standardized prior to analysis to facilitate comparison. Boxes represent point estimates of effects. Box sizes are drawn proportional to the precision of the estimates. Lines represent 95% confidence intervals.

GWASs in UKBB revealed a single genome-wide significant (p < 5 × 10^−8^) locus at 6q16.3 (lead variant rs57989773, EAF_UKBB_ [C-allele] = 0.24; OR 1.23; p = 3.0 × 10^−11^). Meta-analysis with estimates from PHB (OR 1.20; p = 9.84 × 10^−5^) and EGCUT (OR 1.08; p = 0.16) yielded a pooled meta-analysis OR 1.20; p = 5.71 × 10^−14^ (heterogeneity p value = 0.17; Figures 1A–1C). Meta-analysis of all variants yielded no further genome-wide loci. Meta-analysis of our results with previously suggested ED-associated variants also did not result in any further significant loci (Supplemental Material and Methods; Table S3), nor did X chromosome analysis in UKBB.

The association of rs57989773 was consistent across clinically and therapy defined ED, as well as across different ED drug classes (Figures 1C and S1). No further genome-wide significant loci were identified for ED when limited to clinically or therapy defined case subjects (2,032 and 4,142 case subjects, respectively).

A PheWAS of 105 predefined traits (Table S4) using the lead ED SNP rs57989773 found associations with 12 phenotypes at a p value < 5 × 10^−4^ (surpassing the Bonferroni-corrected threshold of 0.05/105), including adiposity (nine traits), adult height, and sleep-related traits. Sex-stratified analyses revealed sexual dimorphism for waist-hip ratio (WHR; unadjusted and adjusted for body mass index) and systolic and diastolic blood pressure (Figure 1D; Table S5).

The lead variant at the 6q16.3 locus, rs57989773, lies in the intergenic region between *MCHR2* and *SIM1*, with *MCHR2* being the closest gene (distances to transcription start sites of 187 kb for *MCHR2* and 284 kb for *SIM1*). Conditional and joint analysis (Supplemental Material and Methods) revealed no secondary, independent signals in the locus. Previous work has implicated the *MCHR2-SIM1* locus in sex-specific associations on age at voice-breaking and menarche.^5^ The puberty timing-associated SNP in the *MCHR2-SIM1* region (rs9321659; ∼500 kb from rs57989773) was not in LD with our lead variant (r^2^ = 0.003, D’ = 0.095) and was not associated with ED (p = 0.32) in our meta-analysis, suggesting that the ED locus represents an independent signal.

**Figure 2:**
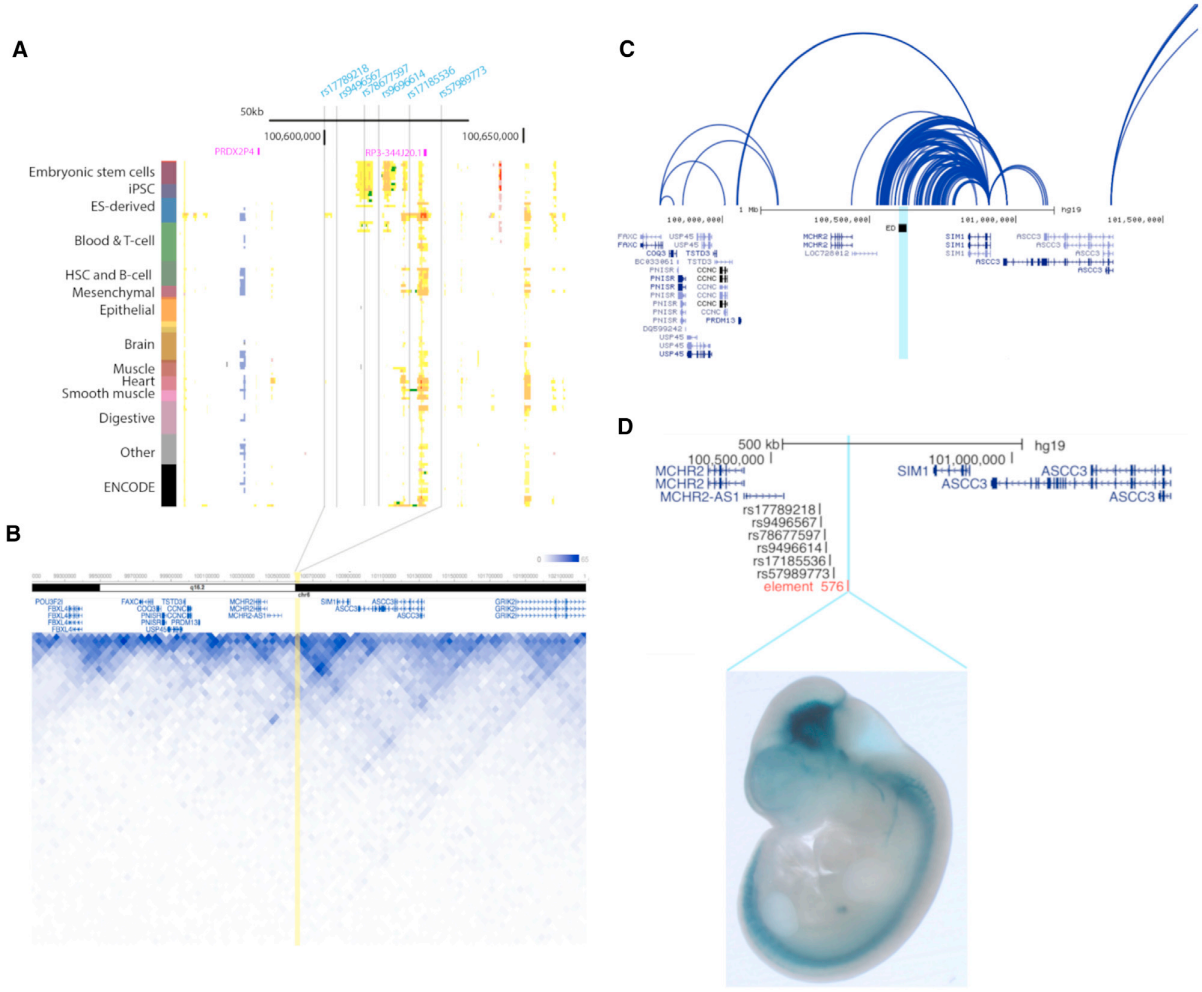
Functional Analysis of 6q16.3 Implicates *SIM1* in ED Pathogenesis (A) ED-associated signal overlaps regulatory annotations in embryonic stem cells. Chromatin state annotations for the ED-associated region across 127 reference epigenomes (rows) for cell and tissue types profiled by the Roadmap Epigenomics Project.^6^, ^7^ Grey vertical lines indicate the position of the ED-associated variant (rs57989773) and its proxies that are in LD r^2^ > 0.8 determined using HaploReg v4.1^34^ (rs17789218, rs9496567, rs78677597, rs9496614, and rs17185536). The lead variant is in proximity to “RP3-344J20.1,” an arginase 2 processed pseudogene (LOC100129854). (B) The ED-associated interval is functionally connected to *SIM1* in embryonic stem cells. The 3D Genome Browser^9^ was used to visualize chromosome conformation capture (Hi-C) interactions contact probabilities in human embryonic stem cells,^8^ revealing high contact probability between the ED-associated region (highlighted in yellow) and *SIM1* at 40-kb resolution. The heatmap values on a color scale correspond to the number of times that reads in two 40-kb bins were sequences together (blue, stronger interaction; white, little or no interaction). (C) The *MCHR2-SIM1* intergenic region forms functional connections to the *SIM1* promoter in endothelial progenitors. The 3D Genome Browser^9^ was used to visualize Capture Hi-C in endothelial precursors.^35^ Light blue vertical line indicates position of the ED-associated interval. (D) The *MCHR2-SIM1* intergenic region harbors a neuronal enhancer. Top: position of human element hs576 (blue vertical line) and the ED-associated variant rs57989773 and its five proxies in r^2^ > 0.8 (rs17789218, rs9496567, rs78677597, rs9496614, rs17185536). hs576 is flanked by genes *MCHR2-AS1* and *SIM1.* This panel was generated using the UCSC genome browser.^36^ Bottom: expression pattern of human element hs576 in a mouse embryo at e11.5. Expression pattern shows that hs576 drives *in vivo* enhancer activity specifically in mesencephalon (midbrain) and cranial nerve. Embryo image was obtained from the VISTA enhancer browser, with permission from the investigators.^10^

To identify the tissue and cell types in which the causal variant(s) for ED may function, we examined chromatin states across 127 cell types^6^, ^7^ for the lead variant rs57989773 and its proxies (r^2^ > 0.8, determined using HaploReg v.4.1) (Supplemental Material and Methods). Enhancer marks in several tissues, including embryonic stem cells, mesenchymal stem cells, and endothelial cells, indicated that the ED-associated interval lies within a regulatory locus (Figure 2A; Table S6).

To predict putative targets and causal transcripts, we assessed domains of long-range three-dimensional chromatin interactions surrounding the ED-associated interval (Figure 2B). Chromosome conformation capture (Hi-C) in human embryonic stem cells^8^ showed that *MCHR2* and *SIM1* were in the same topologically associated domain (TAD) as the ED-associated variants, with high contact probabilities (referring to the relative number of times that reads in two 40-kb bins were sequenced together) between the ED-associated interval and *SIM1* (Figures 2B and S2). This observation was further confirmed in endothelial precursor cells,^9^ where Capture Hi-C revealed strong connections between the *MCHR2-SIM1* intergenic region and the *SIM1* promoter (Figure 2C), pointing toward *SIM1* as a likely causal gene at this locus.

We next used the VISTA enhancer browser^10^ to examine *in vivo* expression data for non-coding elements within the *MCHR2-SIM1* locus. A regulatory human element (hs576), located 30-kb downstream of the ED-associated interval, seems to drive *in vivo* enhancer activity specifically in the midbrain (mesencephalon) and cranial nerve in mouse embryos (Figure 2D). This long-range enhancer close to ED-associated variants recapitulated aspects of *SIM1* expression (Figure 2D), further suggesting that the ED-associated interval belongs to the regulatory landscape of *SIM1*. Taken together these data suggest that the *MCHR2-SIM1* intergenic region harbors a neuronal enhancer and that *SIM1* is functionally connected to the ED-associated region.

Single-minded homolog 1 (*SIM1*) encodes a transcription factor that is highly expressed in hypothalamic neurons.^11^ Rare variants in *SIM1* have been linked to a phenotype of severe obesity and autonomic dysfunction,^12^, ^13^ including lower blood pressure. A summary of the variant-phenotype associations at the 6q16 locus in human and rodent models is shown in Table S7. Post hoc analysis of association of rs57989773 with autonomic traits showed nominal association with syncope, orthostatic hypotension, and urinary incontinence (Figure S3). The effects on blood pressure and adiposity seen in individuals with rare coding variants in *SIM1* are recapitulated in individuals harboring the common ED-risk variants at the 6q16.3 locus (Figure 1D), suggesting that *SIM1* is the causal gene at the ED-risk locus. SIM1-expressing neurons also play an important role in the central regulation of male sexual behavior as mice that lack the melanocortin receptor 4 (encoded by *MC4R*) specifically in SIM1-expressing neurons show impaired sexual performance on mounting, intromission, and ejaculation.^14^ Thus, hypothalamic dysregulation of *SIM1* could present a potential mechanism for the effect of the *MCHR2-SIM1* locus on ED.

An alternative functional mechanism may be explained by proximity of the lead variant (rs57989773) to an arginase 2 processed pseudogene (LOC100129854), a long non-coding RNA (Figure 2A). RPISeq^15^ predicts that the pseudogene transcript would interact with the ARG2 protein, with probabilities of 0.70–0.77. Arginine 2 is involved in nitric oxide production and has a previously established role in erectile dysfunction.^16^, ^17^ GTEx expression data^18^ demonstrated highest mean expression in adipose tissue, with detectable levels in testis, fibroblasts, and brain. Expression was relatively low in all tissues, however, and there was no evidence that any SNPs associated with the top ED signal were eQTLs for the *ARG2* pseudogene or *ARG2* itself.

As a complementary approach, we also used the Data-driven Expression Prioritized Integration for Complex Traits and GWAS Analysis of Regulatory or Functional Information Enrichment with LD correction (DEPICT and GARFIELD, respectively; Supplemental Material and Methods)^19^, ^20^ tools to identify gene-set, tissue-type, and functional enrichments. In DEPICT, the top two prioritized gene-sets were “regulation of cellular component size” and “regulation of protein polymerization,” whereas the top two associated tissue/cell types were “cartilage” and “mesenchymal stem cells.” None of the DEPICT enrichments reached an FDR threshold of 5% (Tables S8–S10). GARFIELD analyses, which assesses enrichment of GWAS signals in regulatory or functional regions in different cell types, also did not yield any statistically significant enrichments, therefore limiting the utility of these approaches in this case.

ED is recognized to be observationally associated with various cardiometabolic traits and lifestyle factors,^21^, ^22^ including type 2 diabetes mellitus (T2D), hypertension, and smoking. To further evaluate these associations, we first conducted LD score regression^23^, ^24^ to evaluate the genetic correlation of ED with a range of traits. LD score regression identified ED to share the greatest genetic correlation with T2D, limb fat mass, and whole-body fat mass (FDR-adjusted p values < 0.05; Table S11).

Next we performed Mendelian randomization^25^ (MR) analyses to evaluate the potential causal role of nine pre-defined cardiometabolic traits on ED risk (selected based on previous observational evidence linking such traits to ED risk^21^), i.e., T2D, insulin resistance, systolic blood pressure, LDL cholesterol, smoking heaviness, alcohol consumption, body mass index, coronary heart disease, and educational attainment (Tables S12–S15). MR identified genetic risk to T2D to be causally implicated in ED: each 1-log higher genetic risk of T2D was found to increase risk of ED with an OR of 1.11 (95% CI 1.05–1.17, p = 3.5 × 10^−4^, which met our *a priori* Bonferroni-corrected significance threshold of 0.0056 [0.05/9]), with insulin resistance likely representing a mediating pathway^26^ (OR 1.36 per 1 standard deviation genetically elevated insulin resistance, 95% CI 1.01–1.84, p = 0.042). Sensitivity analyses were conducted to evaluate the robustness of the T2D-ED estimate (Figure S5, Table S13), including weighted median analyses (OR 1.12, 95% CI 1.02–1.23, p = 0.0230), leave-one-out analysis for all variants (which indicated that no single SNP in the instrument unduly influenced the overall value derived from the summary IVW estimate^27^), and a funnel plot (showing a symmetrical distribution of single-SNP IV estimates around the summary IVW causal estimate). The MR-Egger regression (intercept p = 0.35) provided no evidence to support the presence of directional pleiotropy as a potential source of confounding.^28^

We also identified a potential causal effect of systolic blood pressure (SBP), with higher SBP being linked to higher risk of ED (MR-Egger OR 2.34 per 1 standard deviation higher SBP, 95% CI 1.26–4.36, p = 0.007, with MR-Egger intercept [p = 0.007] suggesting presence of directional pleiotropy). LDL cholesterol (LDL-C) showed minimal evidence of a causal effect (OR 1.07 per 1 standard deviation higher LDL-C, 95% CI 0.98–1.17, p = 0.113), and there was limited evidence to support a role for smoking heaviness or alcohol consumption (Table S15). Genetic risk of coronary heart disease (CHD) showed weak effects on risk of ED, suggesting that pathways leading to CHD may be implicated in ED (OR 1.08, 95% CI 1.00–1.17, p = 0.061). Further, we identified no causal effects of BMI (using a polygenic score or a single SNP in *FTO*) or education on risk of ED.

Genetic variants may inform drug target validation by serving as a proxy for drug target modulation.^29^ ED is most commonly treated using phosphodiesterase 5 (PDE5) inhibitors such as sildenafil. To identify potential phenotypic effects of PDE5 inhibition (e.g., to predict side effects or opportunities for repurposing), we looked for variants in or around *PDE5A*, encoding PDE5, which showed association with the ED phenotype. Of all 4,670 variants within a 1 Mb window of *PDE5A* (chromosome 4:119,915,550–121,050,146 as per GRCh37/hg19), the variant with the strongest association was rs115571325, 26 kb upstream of *PDE5A* (OR_Meta_ 1.25, nominal p value = 8.46 × 10^−4^; Bonferroni-corrected threshold [0.05/4,670] = 1.07 × 10^−5^; Figure S6). Given the weak association with ED, we did not evaluate this variant in further detail.

We have gained insight into ED, a common condition with substantial morbidity, by conducting a large-scale GWAS and performing several follow-up analyses. By aggregating data from 3 cohorts, including 6,175 ED-affected case subjects of European ancestry, we identified a locus associated with ED, with several lines of evidence suggesting *SIM1*, highly expressed in the hypothalamus, to be the causal gene at this locus. Our findings provide human genetic evidence in support of the key role of the hypothalamus in regulating male sexual function.^14^, ^30^, ^31^, ^32^, ^33^

Mendelian randomization implicated risk of T2D as a causal risk factor for ED with suggestive evidence for insulin resistance and systolic blood pressure, corroborating well-recognized observational associations with these cardiometabolic traits.^22^ Further research is needed to explore the extent to which drugs used in the treatment of T2D might be repurposed for the treatment of ED. Lack of evidence for a causal effect of BMI on ED risk in MR analysis (using multiple SNPs across the genome) suggests that the association of the lead SNP (rs57989773) with BMI arises from pleiotropy and that the association of this variant with ED risk is independent of its association with adiposity.

In conclusion, in a large-scale GWAS of more than 6,000 ED-affected case subjects, we provide insights into the biological underpinnings of ED and have elucidated causal effects of various risk factors, including pathways involved in the etiology of T2D. Further large-scale GWASs of ED are needed in order to provide additional clarity on its genetic architecture and etiology and to shed light on potential new therapies.

### Data Availability

Full summary statistics of the erectile dysfunction genome-wide meta-analysis are available at the following URL: http://www.geenivaramu.ee/tools/ED_AJHG_Bovijn_et_al_2018.gz and at the LD Hub GWAShare Center at the following URL: http://ldsc.broadinstitute.org/gwashare/.

## Declaration of Interests

M.N.W. has received speaker fees from Ipsen and Merck. B.N. is a member of the scientific advisory board of Deep Genomics and Consultant for Avanir Therapeutics. M.V.H. has collaborated with Boehringer Ingelheim in research, and in accordance with the policy of the Clinical Trial Service Unit and Epidemiological Studies Unit (University of Oxford), did not accept any personal payment.

## Web Resources

2SampleMR v0.3.4 (R package), https://github.com/MRCIEU/TwoSampleMR
BOLT-LMM v2.3, https://data.broadinstitute.org/alkesgroup/BOLT-LMM/
EPACTS v3.3.0, https://github.com/statgen/EPACTS
EASYQC v9.2, https://www.uni-regensburg.de/medizin/epidemiologie-praeventivmedizin/genetische-epidemiologie/software/
DEPICT v1, https://github.com/perslab/depict
GARFIELD v2, https://www.ebi.ac.uk/birney-srv/GARFIELD/
GCTA v1.26.0, http://cnsgenomics.com/software/gcta/#Overview
HaploReg, http://www.broadinstitute.org/mammals/haploreg/haploreg.php
LD HUB v1.9.0, http://ldsc.broadinstitute.org/
MendelianRandomization v0.2.2 (R package), https://cran.r-project.org/web/packages/MendelianRandomization/index.html
METAL, http://csg.sph.umich.edu/abecasis/metal/
PLINK v1.9, www.cog-genomics.org/plink/1.9/
RPISeq v1.0, http://pridb.gdcb.iastate.edu/RPISeq/references.php
SNPTEST v2.5.2, https://mathgen.stats.ox.ac.uk/genetics_software/snptest/snptest.html#introduction

## Supplemental Data

Data S1. Tables S1–S15

Document S2. Article plus Supplemental Data

## References

[1] Lewis R.W., Fugl-Meyer K.S., Corona G., Hayes R.D., Laumann E.O., Moreira E.D., Rellini A.H., Segraves T. (2010). Definitions/epidemiology/risk factors for sexual dysfunction. J. Sex. Med..

[2] Fry A., Littlejohns T.J., Sudlow C., Doherty N., Adamska L., Sprosen T., Collins R., Allen N.E. (2017). Comparison of sociodemographic and health-related characteristics of UK biobank participants with those of the general population. Am. J. Epidemiol..

[3] Kelly C.A., Soler-Hampejsek E., Mensch B.S., Hewett P.C. (2013). Social desirability bias in sexual behavior reporting: evidence from an interview mode experiment in rural Malawi. Int. Perspect. Sex. Reprod. Health.

[4] Catania J.A., Gibson D.R., Chitwood D.D., Coates T.J. (1990). Methodological problems in AIDS behavioral research: influences on measurement error and participation bias in studies of sexual behavior. Psychol. Bull..

[5] Day F.R., Bulik-Sullivan B., Hinds D.A., Finucane H.K., Murabito J.M., Tung J.Y., Ong K.K., Perry J.R.B. (2015). Shared genetic aetiology of puberty timing between sexes and with health-related outcomes. Nat. Commun..

[6] Kundaje A., Meuleman W., Ernst J., Bilenky M., Yen A., Heravi-Moussavi A., Kheradpour P., Zhang Z., Wang J., Ziller M.J., Roadmap Epigenomics Consortium (2015). Integrative analysis of 111 reference human epigenomes. Nature.

[7] Ernst J., Kellis M. (2015). Large-scale imputation of epigenomic datasets for systematic annotation of diverse human tissues. Nat. Biotechnol..

[8] Dixon J.R., Jung I., Selvaraj S., Shen Y., Antosiewicz-Bourget J.E., Lee A.Y., Ye Z., Kim A., Rajagopal N., Xie W. (2015). Chromatin architecture reorganization during stem cell differentiation. Nature.

[9] Wang Y., Zhang B., Zhang L., An L., Xu J., Li D., Choudhary M.N.K., Li Y., Hu M., Hardison R. (2017). The 3D Genome Browser: a web-based browser for visualizing 3D genome organization and long-range chromatin interactions. bioRxiv.

[10] Visel A., Minovitsky S., Dubchak I., Pennacchio L.A. (2007). VISTA Enhancer Browser--a database of tissue-specific human enhancers. Nucleic Acids Res..

[11] Holder J.L., Zhang L., Kublaoui B.M., DiLeone R.J., Oz O.K., Bair C.H., Lee Y.-H., Zinn A.R. (2004). Sim1 gene dosage modulates the homeostatic feeding response to increased dietary fat in mice. Am. J. Physiol. Endocrinol. Metab..

[12] Ramachandrappa S., Raimondo A., Cali A.M.G., Keogh J.M., Henning E., Saeed S., Thompson A., Garg S., Bochukova E.G., Brage S. (2013). Rare variants in single-minded 1 (SIM1) are associated with severe obesity. J. Clin. Invest..

[13] Michaud J.L., Boucher F., Melnyk A., Gauthier F., Goshu E., Lévy E., Mitchell G.A., Himms-Hagen J., Fan C.M. (2001). Sim1 haploinsufficiency causes hyperphagia, obesity and reduction of the paraventricular nucleus of the hypothalamus. Hum. Mol. Genet..

[14] Semple E., Hill J.W. (2018). Sim1 neurons are sufficient for MC4R-mediated sexual function in male mice. Endocrinology.

[15] Muppirala U.K., Honavar V.G., Dobbs D. (2011). Predicting RNA-protein interactions using only sequence information. BMC Bioinformatics.

[16] Cox J.D., Kim N.N., Traish A.M., Christianson D.W. (1999). Arginase-boronic acid complex highlights a physiological role in erectile function. Nat. Struct. Biol..

[17] Lacchini R., Muniz J.J., Nobre Y.T.D.A., Cologna A.J., Martins A.C.P., Tanus-Santos J.E. (2015). Relationship between Arginase 1 and Arginase 2 levels and genetic polymorphisms with erectile dysfunction. Nitric Oxide.

[18] GTEx Consortium (2013). The Genotype-Tissue Expression (GTEx) project. Nat. Genet..

[19] Pers T.H., Karjalainen J.M., Chan Y., Westra H.-J., Wood A.R., Yang J., Lui J.C., Vedantam S., Gustafsson S., Esko T., Genetic Investigation of ANthropometric Traits (GIANT) Consortium (2015). Biological interpretation of genome-wide association studies using predicted gene functions. Nat. Commun..

[20] Iotchkova V., Ritchie G.R.S., Geihs M., Morganella S., Min J.L., Walter K., Timpson N.J., Dunham I., Birney E., UK10K Consortium (2016). GARFIELD - GWAS Analysis of Regulatory or Functional Information Enrichment with LD correction. bioRxiv.

[21] Selvin E., Burnett A.L., Platz E.A. (2007). Prevalence and risk factors for erectile dysfunction in the US. Am. J. Med..

[22] Yafi F.A., Jenkins L., Albersen M., Corona G., Isidori A.M., Goldfarb S., Maggi M., Nelson C.J., Parish S., Salonia A. (2016). Erectile dysfunction. Nat. Rev. Dis. Primers.

[23] Bulik-Sullivan B.K., Loh P.-R., Finucane H.K., Ripke S., Yang J., Patterson N., Daly M.J., Price A.L., Neale B.M., Schizophrenia Working Group of the Psychiatric Genomics Consortium (2015). LD Score regression distinguishes confounding from polygenicity in genome-wide association studies. Nat. Genet..

[24] Bulik-Sullivan B., Finucane H.K., Anttila V., Gusev A., Day F.R., Loh P.-R., Duncan L., Perry J.R., Patterson N., Robinson E.B., ReproGen Consortium, Psychiatric Genomics Consortium, Genetic Consortium for Anorexia Nervosa of the Wellcome Trust Case Control Consortium 3 (2015). An atlas of genetic correlations across human diseases and traits. Nat. Genet..

[25] Smith G.D., Ebrahim S. (2003). ‘Mendelian randomization’: can genetic epidemiology contribute to understanding environmental determinants of disease?. Int. J. Epidemiol..

[26] Wang Q., Holmes M.V., Davey Smith G., Ala-Korpela M. (2017). Genetic support for a causal role of insulin resistance on circulating branched-chain amino acids and inflammation. Diabetes Care.

[27] Davies N.M., Holmes M.V., Davey Smith G. (2018). Reading Mendelian randomisation studies: a guide, glossary, and checklist for clinicians. BMJ.

[28] Holmes M.V., Ala-Korpela M., Smith G.D. (2017). Mendelian randomization in cardiometabolic disease: challenges in evaluating causality. Nat. Rev. Cardiol..

[29] Walker V.M., Davey Smith G., Davies N.M., Martin R.M. (2017). Mendelian randomization: a novel approach for the prediction of adverse drug events and drug repurposing opportunities. Int. J. Epidemiol..

[30] Argiolas A., Melis M.R. (2005). Central control of penile erection: role of the paraventricular nucleus of the hypothalamus. Prog. Neurobiol..

[31] Argiolas A., Melis M.R. (2013). Neuropeptides and central control of sexual behaviour from the past to the present: a review. Prog. Neurobiol..

[32] Courtois F., Carrier S., Charvier K., Guertin P.A., Journel N.M. (2013). The control of male sexual responses. Curr. Pharm. Des..

[33] Giuliano F., Rampin O. (2004). Neural control of erection. Physiol. Behav..

[34] Ward L.D., Kellis M. (2012). HaploReg: a resource for exploring chromatin states, conservation, and regulatory motif alterations within sets of genetically linked variants. Nucleic Acids Res..

[35] Javierre B.M., Burren O.S., Wilder S.P., Kreuzhuber R., Hill S.M., Sewitz S., Cairns J., Wingett S.W., Várnai C., Thiecke M.J., BLUEPRINT Consortium (2016). Lineage-specific genome architecture links enhancers and non-coding disease variants to target gene promoters. Cell.

[36] Casper J., Zweig A.S., Villarreal C., Tyner C., Speir M.L., Rosenbloom K.R., Raney B.J., Lee C.M., Lee B.T., Karolchik D. (2018). The UCSC Genome Browser database: 2018 update. Nucleic Acids Res..

