# Supplementary material for: GWAS Identifies Risk Locus for Erectile Dysfunction and Implicates Hypothalamic Neurobiology and Diabetes in Etiology
Source: Am J Hum Genet. 2018 Dec 21;104(1):157–63. doi: 10.1016/j.ajhg.2018.11.004 (PMC6323625; doi:10.1016/j.ajhg.2018.11.004)
Supplement: Document S1. Figures S1–S7 and Supplemental Material and Methods [file mmc1.pdf]

The American Journal of Human Genetics, Volume *104*

Supplemental Data

GWAS Identifies Risk Locus for Erectile Dysfunction and Implicates Hypothalamic Neurobiology and Diabetes in Etiology

Jonas Bovijn, Leigh Jackson, Jenny Censin, Chia-Yen Chen, Triin Laisk, Samantha Laber, Teresa Ferreira, Sara L. Pulit, Craig A. Glastonbury, Jordan W. Smoller, Jamie W. Harrison, Katherine S. Ruth, Robin N. Beaumont, Samuel E. Jones, Jessica Tyrrell, Andrew R. Wood, Michael N. Weedon, Reedik Mägi, Benjamin Neale, Cecilia M. Lindgren, Anna Murray, and Michael V. Holmes

Figure S1

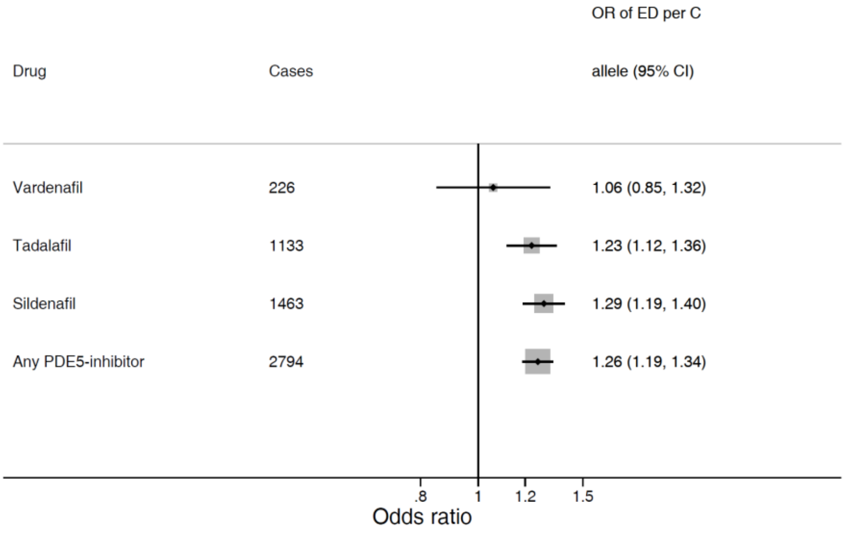

The association of rs57989773 remains consistent across different ED drug classes.

Figure S2

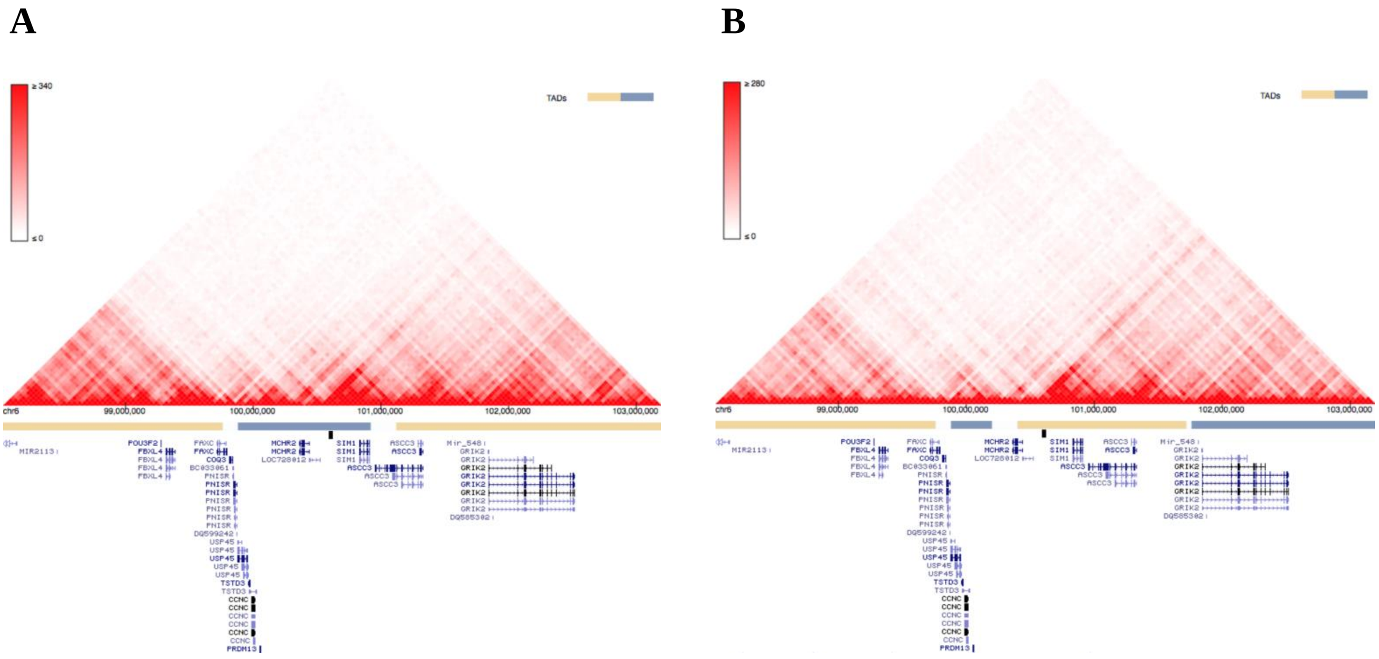

Hi-C interaction maps in several cell types. The 3D Genome Browser[1] was used to visualize the spatial organisation surrounding the ED-associated region. Heatmap shows chromosome conformation capture (Hi-C) interactions contact probabilities in (A) human MES mesendoderm cells[2] at 40-kb resolution; and (B) human mesenchymal stem cells (MSC)[2] at 40-kb resolution. The heat map values on a colour scale correspond to the number of times that reads in two 40-kb bins were sequences together (red - stronger interaction, white - little or no interaction). The second panel indicates the location of the ED-associated region. The third panel shows the UCSC reference genes.

Figure S3

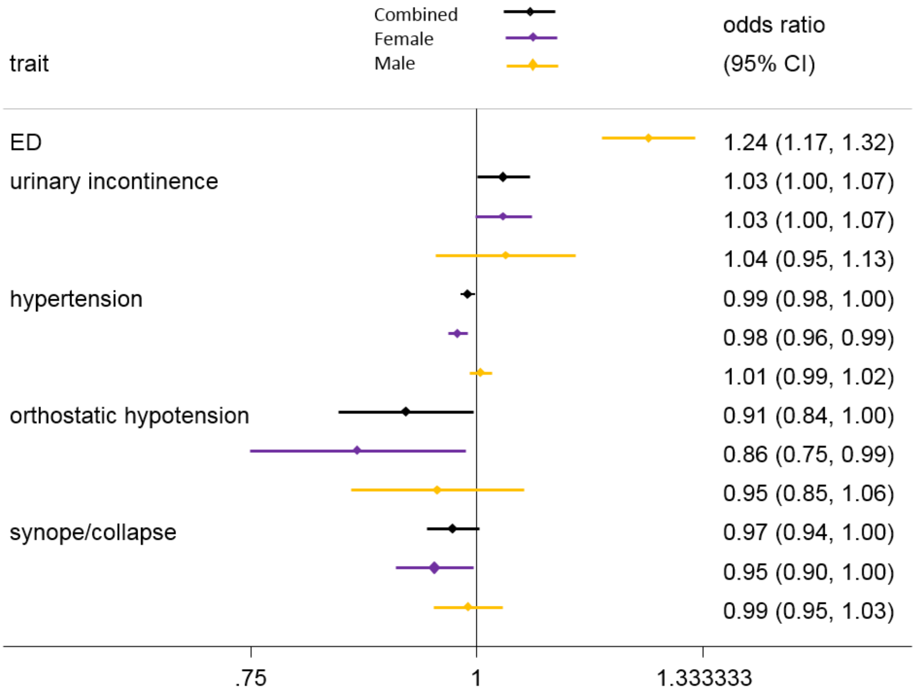

Association of rs57989773 with autonomic phenotypes.

Figure S4

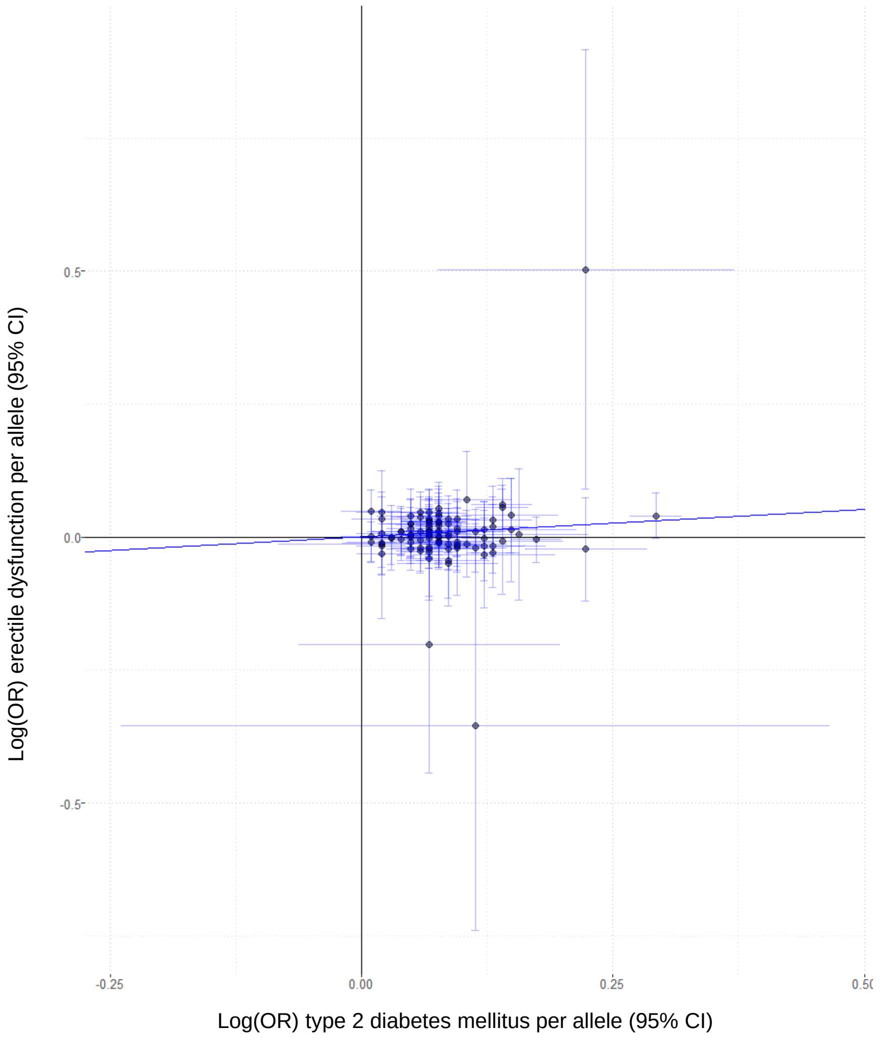

Association of individual SNPs (N=103) with risk for type 2 diabetes mellitus and erectile dysfunction

The blue line represents the inverse variance weighted (IVW) estimate.

Figure S5

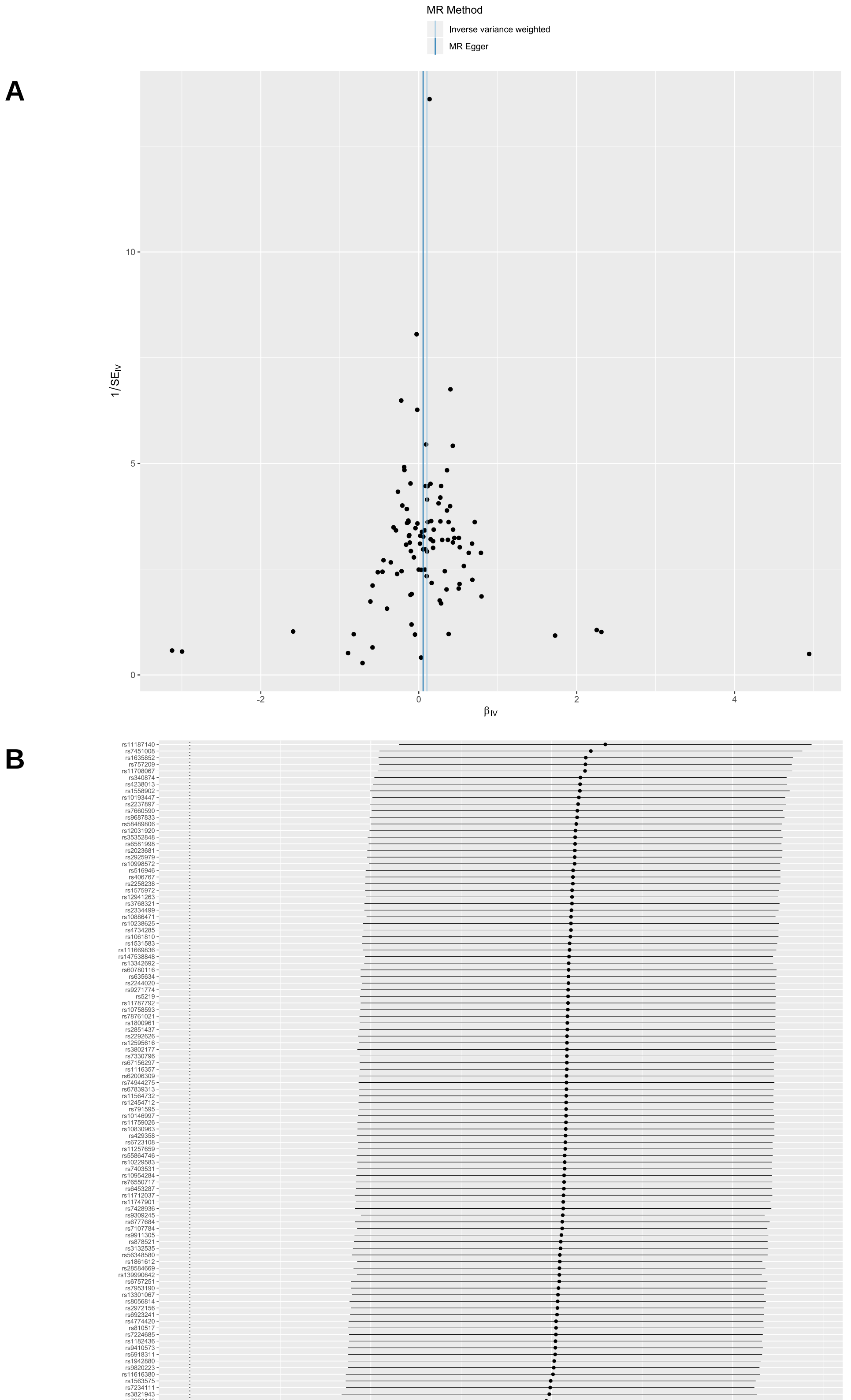

Sensitivity analyses for type 2 diabetes mellitus – erectile dysfunction MR analysis

A. Symmetrical funnel plot of single SNP causal estimates, suggesting absence of directional pleiotropy.

B. Forest plot of leave-one-out analysis (Y-axis indicating which variant was left out for each analysis) indicating that no single SNP in the instrument altered the significance of the IVW estimate (all IVW estimates p-values < 0.0056)

Figure S6

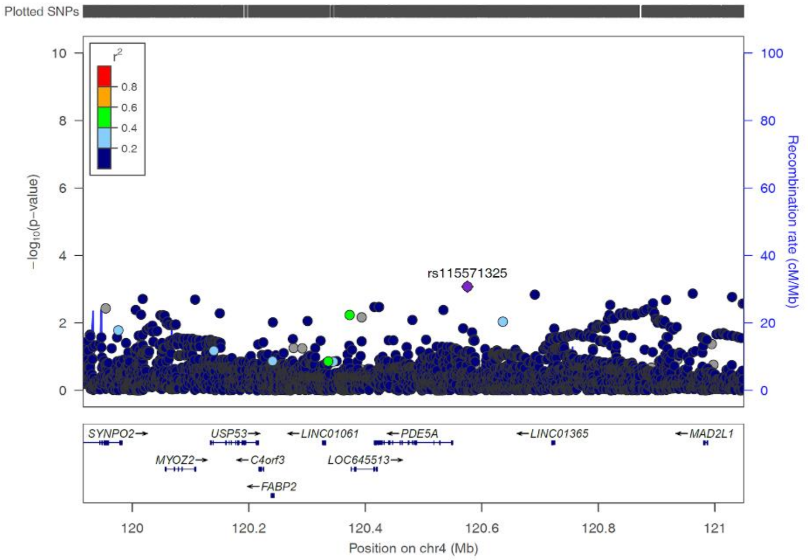

Association of variants in the PDE5A region with ED.

Figure S7

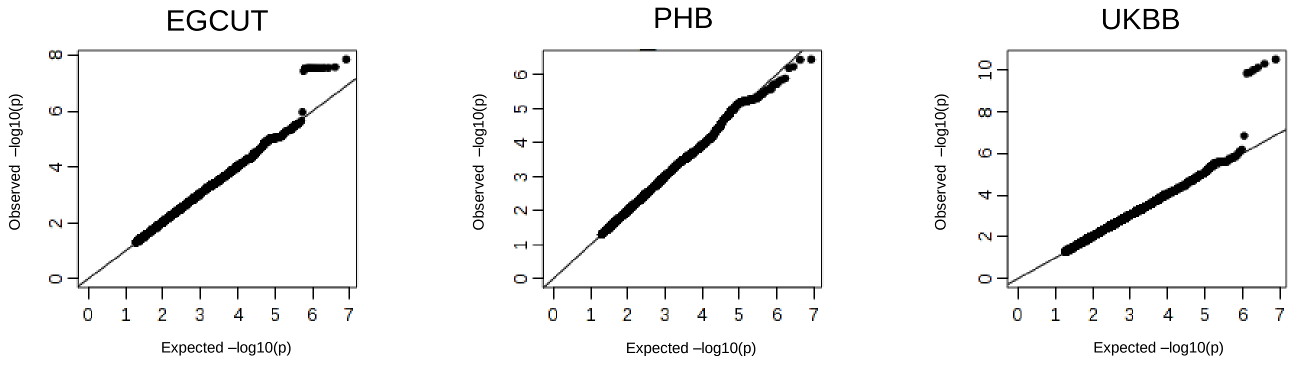

Quantile-quantile (Q-Q) plot of observed versus expected P values in each cohort

Supplemental Material and Methods

STUDY SUBJECTS

Study subjects: Partners HealthCare Biobank

We identified cases of erectile dysfunction (ED) and healthy male controls from the Partners HealthCare Biobank,[3,4] a biorepository of consented patient samples at Partners HealthCare (the parent organization of Massachusetts General Hospital and Brigham and Women's Hospital). All patients who participate in the Partners Biobank are consented for their samples to be linked to their identified clinical information.

The ED cases (Table S1) were identified by querying the Partners Biobank with ICD-10 code N52 (male erectile dysfunction). In addition to ICD-10 code, we also identified ED cases by querying the Partners Biobank with the following drug prescriptions: sildenafil, viagra (25mg, 50mg, and 100mg tablet), tadalafil, cialis (10mg and 20mg tablet), vardenafil, levitra (5mg, 10mg, and 20mg tablet). Given the overlap in indication for phosphodiesterase-5a inhibitors (such as sildenafil and tadalafil), patients with pulmonary hypertension (identified using ICD-10 codes I27.0 and I27.2) were excluded from the identified ED case pool. The controls were males from the Partners Biobank. We also extracted age for ED cases and controls from the Biobank for subsequent analyses. There are 1,943 ED cases and 5,723 male controls with genome-wide genotyped data.

Study subjects: UK Biobank

UK Biobank (UKBB) is a prospective study of more than 500,000 British individuals recruited from 2006 to 2010, aged between 45 and 69.[5] Phenotypic information available includes self-reported medical history (including medication use) as ascertained by verbal interview at enrolment and hospital-derived electronic health record (EHR) data, including International Classification of Disease (ICD-10) diagnosis codes and Office of Population and Censuses

Surveys (OPCS-4) procedure codes.

Individuals in UKBB were defined as having ED (Table S1) on the basis of at least one of the following criteria: Self-reported ED/impotence at time of enrolment; hospitalisation for ICD-10 codes N48.4, F52.2 or N52; hospitalisation for OPCS-4 coded procedures L97.1 or N32.6; or self-reported ED medication (either sildenafil, Viagra, tadalafil, Cialis, vardenafil, Levitra) use. Patients with pulmonary hypertension were excluded from the analyses (identified using ICD-10 codes I27.0 and I27.2, and OPCS-4 codes X821, X822, X823, X824).

Of the 488,377 individuals with available genotype data, we excluded individuals that had: non-white or mixed self-reported ethnicity at any point during follow-up, withdrawn their consent for participation; high sample heterozygosity and missingness; >10 third degree relatives; putative sex chromosome aneuploidy; sex mismatches (genetic vs. self-reported and between assessments); ethnicity mismatches (genetic vs self-reported for White British individuals, and mismatches between assessments) and codes for pulmonary hypertension. After applying these filters, 199,352 male subjects remained, of whom 3,050 met the case-definition criteria for ED, whereas 196,302 subjects served as controls.

Study subjects: Estonian Genome Center of the University of Tartu

The Estonian Genome Center of the University of Tartu (EGCUT) is a population-based biobank with a current cohort size of 51,515 participants.[6] Upon recruitment, the biobank participants filled out a thorough questionnaire, covering lifestyle, diet and clinical diagnoses (described by ICD-10 codes). Data are periodically updated by linking with national health registries. In EGCUT, ED cases (Table S1) were defined using ICD-10 codes F52.2 or N48.4; or data on prescribed drugs (with active compounds tadalafil, sildenafil, vardenafil). Males without any of these diagnosis codes or prescribed drugs were used as controls. The analysis included a total of 1,182 male cases and 15,605 male controls.

GENOTYPING, QC, AND IMPUTATION

Genotyping, QC, and imputation: Partners Healthcare Biobank

DNA samples from the patients in the Partners Biobank were extracted from whole blood. A total of 20,087 samples were genotyped with Illumina Multi-Ethnic Genotyping Array (first batch), Expanded Multi-Ethnic Genotyping Array (second batch), and Multi-Ethnic Global BeadChip (third batch), all of which were designed to capture the global diversity of genetic backgrounds. The number of genotyped variants ranged from 1,416,020 to 1,778,953. We performed QC on each genotyping batch separately as follows: we removed single nucleotide polymorphisms (SNPs) with genotype missing rate > 0.05 before sample-based QC; excluded samples with genotype missing rate > 0.02, absolute value of heterozygosity > 0.2, or failed sex checks; removed SNPs with missing rate > 0.02 after sample-based QC. To merge genotyping batches for imputation and analyses, we performed batch QC by removing SNPs with significant batch association (p-value < 1.0×10^{-6} between different batches). Since the Partners Biobank samples have diverse population backgrounds, we performed Hardy-Weinberg equilibrium test (p-value < 1.0×10^{-6}) for SNP-based QC after extracting samples with European ancestry (see below). We also performed relatedness tests by identifying pairs of samples with π > 0.2 and excluding one sample from each related sample pair (560 samples excluded). All QC were conducted using PLINK v1.9 and R software.

We extracted samples with European ancestry based on principal component analysis (PCA) with 1000 Genomes Project reference samples. Details of the procedure used to extract European ancestry samples were described previously.[7,8] Briefly, we ran PCA on study samples combined with 1000 Genomes Project reference samples and calculate Euclidean distance (d_{EUR}) for each study sample to the average PC1 and PC2 of the 1000 Genomes Project EUR samples. A total of 16,453 study samples with European ancestry were extracted based on d_{EUR} < 0.003. We then performed PCA on the European ancestry samples to obtain PCs for the subsequent analyses.

Genotype imputation was performed on the QCed European ancestry samples with a 2-step pre-phasing/imputation approach. We used Eagle2 for the pre-phasing and minimac3 for imputation, with a reference panel from 1000 Genomes Project phase 3. The final analytic data includes 1,943 ED cases and 5,723 controls of European ancestry with imputed genotype data.

Genotyping, QC, and imputation: UK Biobank

Genotyping, quality control and imputation were performed centrally by UKBB, and details are described elsewhere[9] (see also http://biobank.ctsu.ox.ac.uk/crystal/label.cgi?id=100319). Briefly, genotype data are available for 488,377 individuals, 49,950 of whom were genotyped using the Applied Biosystems™ UK BiLEVE Axiom™ Array by Affymetrix (containing 807,411 markers[10]). The remaining 438,427 individuals were genotyped using the Applied Biosystems™ UK Biobank Axiom™ Array by Affymetrix (containing 825,927 markers). Both of these arrays were specifically designed for use in the UKBB project and share ~95% of marker content. Phasing was done using SHAPEIT3, and imputation was conducted using IMPUTE4. For imputation, the Haplotype Reference Consortium (HRC) panel[11] was used wherever possible, and for SNPs not in that reference panel, a merged UK10K + 1000 Genomes reference panel was used. SNPs were imputed from both panels, but the HRC imputation was preferentially used for SNPs present in both panels. Given known issues with non-HRC imputed SNPs with the current UKBB genotype data release (http://www.ukbiobank.ac.uk/2017/07/important-note-about-imputed-genetics-data/), we included only HRC-imputed SNPs in our dataset.

Genotyping, QC, and imputation: Estonian Genome Center of the University of Tartu

In EGCUT, DNA was extracted from whole blood. Genotyping was carried out using Illumina Human CoreExome, OmniExpress, 370CNV BeadChip and GSA arrays. Genotype array data

was filtered sample-wise by excluding on the basis of call rate (<98%), heterozygosity (>mean±3 SD), genotype and phenotype sex discordance, cryptic relatedness (IBD>20%) and outliers from the European descent based on the MDS plot in comparison with HapMap reference samples. SNP quality filtering included call rate (<99%), MAF (<1%) and extreme deviation from Hardy–Weinberg equilibrium (P-value$<1 \times 10^{-4}$). Imputation was performed on the QCed samples, using SHAPEIT2 for prephasing, the Estonian-specific reference panel[12] and IMPUTE2 with default parameters.

STATISTICAL ANALYSES

Each cohort conducted association analysis using locally supported methods and workflows. Each analysis was adjusted for age, with additional adjustment for principal components (to adjust for population stratification) if logistic regression was used (PHB only). Linear mixed model-based methods (as used in UKBB and EGCUT) do not require the inclusion of principal components to adjust for population stratification[13].

Statistical analyses: Partners Healthcare Biobank

Logistic regression was used to test genome-wide association for ED in PHB, adjusted for 10 PCs and age, using PLINK v1.9[14].

Statistical analyses: UK Biobank

We used BOLT-LMM[13] v2.3 to perform association analyses in UKBB. BOLT-LMM computes statistics for testing association between phenotype and genotypes using a linear mixed model (LMM)[13]. The performance of BOLT-LMM on the UKBB dataset has been previously validated[14], allows for inclusion of related individuals and has been shown to significantly

increase power when compared to traditional linear regression methods[15]. All analyses were adjusted for age.

Statistical analyses: Estonian Genome Center of the University of Tartu

EPACTS v3.3.0 (using option q.emmax) was used to perform association testing in EGCUT, adjusting for age at recruitment and the kinship matrix.

Statistical analyses: Meta-analyses

Prior to meta-analysis, we performed standardized study-level quality control using EASYQC[16]. All three studies (UKBB, PHB, and EGCUT) were subjected to the same QC measures, with inclusion of variants with imputation INFO scores > 0.4 and MAF > 1%. Indels and CNVs were not included in the meta-analysis. Genomic control (GC) correction was applied to each dataset prior to meta-analysis (Pre-correction GC lambda values: UKBB = 1.047; PHB = 1.01; EGCUT = 1.006).

We confirmed that the lead variant at 6q16.3, rs57989773, had acceptable imputation quality in all cohorts (UKBB INFO score: 0.94; PHB INFO score: 0.89; EGCUT INFO score: 0.92). Quantile-quantile (QQ) plots for each cohort are shown in Figure S7.

METAL software[17] was used for performing fixed effect inverse-variance weighted meta-analysis of allelic effect sizes after conversion onto the log-odds scale for the LMM-derived estimates from UKBB and EGCUT. This has been shown to be a valid method for meta-analysis of GWA studies of binary phenotypes using linear mixed models[18].

In addition to the main meta-analysis, we conducted two additional meta-analyses using clinically- or therapy-defined cases respectively. Since only UKBB and PHB had this data available, only these two cohorts were included in these analyses. Identical methodology as described above was followed when performing these meta-analyses.

Statistical analyses: Conditional and joint analysis

We performed conditional and joint analysis of the *MCHR2-SIM1* locus using GCTA[19]. We used individual-level genotype data from UKBB as a reference sample for LD (excluding SNPs with missingness >5%, imputation info score <0.3 and MAF <0.01%, and excluding individuals as for the GWAS analysis in UKBB detailed above). Using relatedness data provided by the UKBB data-release, we reviewed pairwise genetic relationships between individuals and removed one of each pair of individuals with an estimated relatedness of >0.025.

Replication and meta-analysis of previously reported ED-associated SNPs

We performed a literature review for previous ED GWAS and identified three studies[20-22]. We extracted all independent, autosomal SNPs associated with ED with $p \leq 9 \times 10^{-6}$ (i.e. all autosomal SNPs included on the GWAS Catalog database entry for "impotence" or "erectile dysfunction"), yielding 23 SNPs. Summary statistics for these variants were subsequently extracted from our GWAMA results. We then performed effective sample-size weighted Z-score meta-analyses of previously reported summary statistics for 17 variants (3 variants omitted due to MAF < 1% in all three cohorts in our study, 2 variants omitted due to absence of effect size direction in the original report and 1 due to alleles being inconsistent between studies) with summary statistics extracted from our GWAMA (Table S3).

IN-SILICO FUNCTIONAL FOLLOW-UP

UCSC Genome Browser (available at http://genome.ucsc.edu/; December 2013 (GRCh37/hg19) assembly) was used to determine distance of rs57989773 to the transcription start sites of *MCHR2* and *SIM1*.

Phenome-wide Association Scan (PheWAS)

A PheWAS of traits in UKBB was carried out as previously described[23]. Briefly, a range of phenotypes were available in UK Biobank, derived from self-reported questionnaire data, ICD10 diagnoses and baseline measurements at clinic visits as part of the study. We tested the association of the lead ED SNP rs57989773 with a range of phenotypes and traits including: anthropometric, reproductive, cardiovascular, learning/memory and incidence of various diseases (Table S4). Association testing was carried out with inverse normalised phenotypes to account for any skewed distributions, using linear regression models in STATA 13, adjusting for SNP chip type (UKB Axiom or UK BiLEVE), ancestry-principal components 1 to 5 supplied by UK Biobank, test centre and age (or year of birth for age at menarche) with the exception of three traits: hypertension, hypothyroidism and household income. Hypertension and hypothyroidism were tested using logistic regression and household income was tested using ordinal logistic regression. The logistic and ordinal logistic models were adjusted using the same covariates as the linear regression model. Due to the nature of the ED phenotype and previously reported sex-specific effects in the *MCHR2-SIM1* locus, we performed sex-specific analyses on significant traits. Female samples in UKBB were ascertained using self-reported sex, where such reported sex was consistent with genetically determined sex. Samples with discordant self-reported/chromosomal sex, were not included in any analyses. Testing for heterogeneity was performed to assess whether any observed sex-specific effects were significant after accounting for multiple testing.

DEPICT

DEPICT[24] (Data-driven Expression Prioritized Integration for Complex Traits) is a comprehensive pathway analysis tool. We used DEPICT to prioritise likely causal genes at associated loci, and to identify enriched gene sets and tissue and cells types where genes from prioritised loci are highly expressed.

In our study, independent variants were identified in the genome-wide association meta-analysis result using PLINK v.1.9 to clump SNPs at an LD-threshold of r^2=0.1 and a physical distance threshold of 500kb, resulting in 37 independent variants with a p-value threshold of $p < 1 \times 10^{-5}$. SNPs in HLA regions, on sex chromosomes or not present in 1000 Genomes Project were excluded from DEPICT analysis. DEPICT was used to identify tissue and cell type annotations in which genes from associated regions were highly expressed, to identify reconstituted gene sets enriched for genes from associated regions and to prioritize genes within associated regions.

GARFIELD

GARFIELD is a functional enrichment analysis approach described more fully elsewhere[25]. Briefly, GARFIELD is a nonparametric method to assess enrichment of GWAS signals in regulatory or functional regions in different cell-types. The software LD-prunes the GWAS-data before assessing fold enrichment at different p-value thresholds from the GWAS study of interest. To minimize bias, it takes into account LD-structure, gene density, and allele frequencies.

LD Score regression and cross-trait genetic correlation analysis

LD Hub[26] was used to conduct LD Score regression and cross-trait genetic correlation analysis. LD Hub is a centralized database of summary-level GWAS results for >100 diseases/traits from different publicly available resources/consortia and uses a web interface that automates the LD Score regression and cross-trait genetic correlation analysis pipeline.

LD Score regression[27] quantifies the contribution of true polygenic signal and confounding biases, such as cryptic relatedness and population stratification, to inflated distribution of test

statistics in genome-wide association studies, by examining the relationship between test statistics and linkage disequilibrium (LD). The LD Score regression intercept can be used to estimate a more powerful and accurate correction factor than genomic control.

Genetic correlation analysis was conducting using cross-trait LD Score regression[28]. This is a technique estimating genetic correlation that requires only GWAS summary statistics and is not biased by sample overlap.

Mendelian Randomization

Multiple traits have shown association with ED in observational studies, including BMI, educational attainment, hypertension, diabetes, hypercholesterolemia, smoking and cardiovascular disease[29]. We therefore investigated the causal effects of these traits using Mendelian randomization (MR) (Table S12). All MR analyses were performed in R 3.4.3 (R Development Core Team (2008). R: A language and environment for statistical computing. R Foundation for Statistical Computing, Vienna, Austria. http://www.R-project.org) using the *MendelianRandomization* R package[30]. Instrument rsIDs were updated using Python version 3.5.2 (Python Software Foundation. Python Language Reference, version 3.5.2. http://www.python.org), libraries Pandas[31] and Biopython[32], and the NCBI SNP website (Available from: https://www.ncbi.nlm.nih.gov/snp/). Proxies (r^2 >0.8) were identified using SNiPA[33]. Leave-one-out analyses and the funnel plot were created using the *TwoSampleMR* R package[34].

ETHICAL APPROVAL

All analyses in UKBB were performed under UKBB application number 11867. UKBB received ethical approval from the North West Centre for Research Ethics Committee (reference number 11/NW/0382).

Analyses in EGCUT were approved by the Ethics Review Committee of the University of Tartu (243T-12).

The Partners HealthCare Biobank maintains blood and DNA samples from consented patients seen at Partners HealthCare hospitals in the Boston area of Massachusetts. Patients are recruited in the context of clinical care appointments, and also electronically at Partners HealthCare. Biobank subjects provide consent for the use of their samples and data in broad-based research.

ACKNOWLEDGEMENTS

We gratefully acknowledge all the studies and databases that made GWAS summary data available: ADIPOGen (Adiponectin genetics consortium), C4D (Coronary Artery Disease Genetics Consortium), CARDIoGRAM (Coronary ARtery DIsease Genome wide Replication and Meta-analysis), CKDGen (Chronic Kidney Disease Genetics consortium), dbGAP (database of Genotypes and Phenotypes), DIAGRAM (DIAbetes Genetics Replication And Meta-analysis), ENIGMA (Enhancing Neuro Imaging Genetics through Meta Analysis), EAGLE (EArly Genetics & Lifecourse Epidemiology Eczema Consortium, excluding 23andMe), EGG (Early Growth Genetics Consortium), GABRIEL (A Multidisciplinary Study to Identify the Genetic and Environmental Causes of Asthma in the European Community), GCAN (Genetic Consortium for Anorexia Nervosa), GEFOS (GEnetic Factors for OSteoporosis Consortium), GIANT (Genetic Investigation of ANthropometric Traits), GIS (Genetics of Iron Status consortium), GLGC (Global Lipids Genetics Consortium), GPC (Genetics of Personality Consortium), GUGC (Global Urate and Gout consortium), HaemGen (haemotological and platelet traits genetics consortium), HRgene (Heart Rate consortium), IIBDGC (International Inflammatory Bowel Disease Genetics Consortium), ILCCO (International Lung Cancer Consortium), IMSGC (International Multiple Sclerosis Genetic Consortium), MAGIC (Meta-Analyses of Glucose and Insulin-related traits Consortium), MESA (Multi-Ethnic Study of Atherosclerosis), PGC (Psychiatric Genomics Consortium), Project MinE consortium, ReproGen (Reproductive Genetics Consortium), SSGAC (Social Science Genetics Association Consortium) and

TAG (Tobacco and Genetics Consortium), TRICL (Transdisciplinary Research in Cancer of the Lung consortium), UK Biobank. We gratefully acknowledge the contributions of Alkes Price (the systemic lupus erythematosus GWAS and primary biliary cirrhosis GWAS) and Johannes Kettunen (lipids metabolites GWAS).

References

1. Wang, Y., Zhang, B., Zhang, L., An, L., Xu, J., Li, D., Choudhary, M.N.K., Li, Y., Hu, M., Hardison, R., et al. (2017). The 3D Genome Browser: a web-based browser for visualizing 3D genome organization and long-range chromatin interactions. BioRxiv. doi: https://doi.org/10.1101/112268.

2. Dixon, J.R., Jung, I., Selvaraj, S., Shen, Y., Antosiewicz-Bourget, J.E., Lee, A.Y., Ye, Z., Kim, A., Rajagopal, N., Xie, W., et al. (2015). Chromatin architecture reorganization during stem cell differentiation. Nature *518*, 331–336.

3. Smoller, J.W., Karlson, E.W., Green, R.C., Kathiresan, S., MacArthur, D.G., Talkowski, M.E., Murphy, S.N., and Weiss, S.T. (2016). An eMERGE Clinical Center at Partners Personalized Medicine. J Pers Med *6*.

4. Gainer, V.S., Cagan, A., Castro, V.M., Duey, S., Ghosh, B., Goodson, A.P., Goryachev, S., Metta, R., Wang, T.D., Wattanasin, N., et al. (2016). The Biobank Portal for Partners Personalized Medicine: A Query Tool for Working with Consented Biobank Samples, Genotypes, and Phenotypes Using i2b2. J Pers Med *6*.

5. Collins, R. (2012). What makes UK Biobank special? Lancet *379*, 1173–1174.

6. Leitsalu, L., Haller, T., Esko, T., Tammesoo, M.-L., Alavere, H., Snieder, H., Perola, M., Ng, P.C., Mägi, R., Milani, L., et al. (2015). Cohort Profile: Estonian Biobank of the Estonian Genome Center, University of Tartu. Int. J. Epidemiol. *44*, 1137–1147.

7. Stein, M.B., Chen, C.-Y., Ursano, R.J., Cai, T., Gelernter, J., Heeringa, S.G., Jain, S., Jensen, K.P., Maihofer, A.X., Mitchell, C., et al. (2016). Genome-wide Association Studies of Posttraumatic Stress Disorder in 2 Cohorts of US Army Soldiers. JAMA Psychiatry *73*, 695–704.

8. Chen, C.-Y., Pollack, S., Hunter, D.J., Hirschhorn, J.N., Kraft, P., and Price, A.L. (2013). Improved ancestry inference using weights from external reference panels. Bioinformatics *29*, 1399–1406.

9. Bycroft, C., Freeman, C., Petkova, D., Band, G., Elliott, L.T., Sharp, K., Motyer, A., Vukcevic, D., Delaneau, O., O'Connell, J., et al. (2017). Genome-wide genetic data on ~500,000 UK Biobank participants. BioRxiv. doi: https://doi.org/10.1101/166298.

10. Wain, L.V., Shrine, N., Miller, S., Jackson, V.E., Ntalla, I., Soler Artigas, M., Billington, C.K., Kheirallah, A.K., Allen, R., Cook, J.P., et al. (2015). Novel insights into the genetics of smoking behaviour, lung function, and chronic obstructive pulmonary disease (UK BiLEVE): a genetic association study in UK Biobank. Lancet Respir Med *3*, 769–781.

11. McCarthy, S., Das, S., Kretzschmar, W., Delaneau, O., Wood, A.R., Teumer, A., Kang, H.M., Fuchsberger, C., Danecek, P., Sharp, K., et al. (2016). A reference panel of 64,976 haplotypes for genotype imputation. Nat. Genet. *48*, 1279–1283.

12. Mitt, M., Kals, M., Pärn, K., Gabriel, S.B., Lander, E.S., Palotie, A., Ripatti, S., Morris, A.P., Metspalu, A., Esko, T., et al. (2017). Improved imputation accuracy of rare and low-frequency variants using population-specific high-coverage WGS-based imputation reference panel. Eur. J. Hum. Genet. *25*, 869–876.

13. Chang, C.C., Chow, C.C., Tellier, L.C., Vattikuti, S., Purcell, S.M., and Lee, J.J. (2015). Second-generation PLINK: rising to the challenge of larger and richer datasets. Gigascience *4*, 7.

14. Loh, P.-R., Tucker, G., Bulik-Sullivan, B.K., Vilhjálmsson, B.J., Finucane, H.K., Salem, R.M., Chasman, D.I., Ridker, P.M., Neale, B.M., Berger, B., et al. (2015). Efficient Bayesian mixed-model analysis increases association power in large cohorts. Nat. Genet. *47*, 284–290.

15. Loh, P.-R., Kichaev, G., Gazal, S., Schoech, A.P., and Price, A.L. (2018). Mixed model association for biobank-scale data sets. BioRxiv. doi: https://doi.org/10.1101/194944

16. Winkler, T.W., Day, F.R., Croteau-Chonka, D.C., Wood, A.R., Locke, A.E., Mägi, R., Ferreira, T., Fall, T., Graff, M., Justice, A.E., et al. (2014). Quality control and conduct of genome-wide association meta-analyses. Nat. Protoc. *9*, 1192–1212.

17. Willer, C.J., Li, Y., and Abecasis, G.R. (2010). METAL: fast and efficient meta-analysis of genomewide association scans. Bioinformatics *26*, 2190–2191.

18. Cook, J.P., Mahajan, A., and Morris, A.P. (2017). Guidance for the utility of linear models in meta-analysis of genetic association studies of binary phenotypes. Eur. J. Hum. Genet. *25*, 240–245.

19. Yang, J., Ferreira, T., Morris, A.P., Medland, S.E., Genetic Investigation of ANthropometric Traits (GIANT) Consortium, DIAbetes Genetics Replication And Meta-analysis (DIAGRAM) Consortium, Madden, P.A.F., Heath, A.C., Martin, N.G., Montgomery, G.W., et al. (2012). Conditional and joint multiple-SNP analysis of GWAS summary statistics identifies additional variants influencing complex traits. Nat. Genet. *44*, 369–375.

20. Hotaling, J.M., Waggott, D.R., Goldberg, J., Jarvik, G., Paterson, A.D., Cleary, P.A., Lachin, J., Sarma, A., Wessells, H., and DCCT/EDIC Research Group (2012). Pilot genome-wide association search identifies potential loci for risk of erectile dysfunction in type 1 diabetes using the DCCT/EDIC study cohort. J. Urol. *188*, 514–520.

21. Kerns, S.L., Stock, R., Stone, N., Buckstein, M., Shao, Y., Campbell, C., Rath, L., De Ruysscher, D., Lammering, G., Hixson, R., et al. (2013). A 2-stage genome-wide association study to identify single nucleotide polymorphisms associated with development of erectile dysfunction following radiation therapy for prostate cancer. Int. J. Radiat. Oncol. Biol. Phys. *85*, e21–e28.

22. Kerns, S.L., Ostrer, H., Stock, R., Li, W., Moore, J., Pearlman, A., Campbell, C., Shao, Y., Stone, N., Kusnetz, L., et al. (2010). Genome-wide association study to identify single nucleotide polymorphisms (SNPs) associated with the development of erectile dysfunction in African-American men after radiotherapy for prostate cancer. Int. J. Radiat. Oncol. Biol. Phys. *78*, 1292–1300.

23. Tuke, M.A., Ruth, K.S., Wood, A.R., Beaumont, R.N., Tyrrell, J., Jones, S.E., Yaghootkar, H., Turner, C.L.S., Donohoe, M.E., Brooke, A.M., et al. (2017). Phenotypes associated with female X chromosome aneuploidy in UK Biobank: an unselected, adult, population-based cohort. BioRxiv. doi: https://doi.org/10.1101/177659.

24. Pers, T.H., Karjalainen, J.M., Chan, Y., Westra, H.-J., Wood, A.R., Yang, J., Lui, J.C., Vedantam, S., Gustafsson, S., Esko, T., et al. (2015). Biological interpretation of genome-wide association studies using predicted gene functions. Nat. Commun. *6*, 5890.

25. Iotchkova, V., Ritchie, G.R.S., Geihs, M., Morganella, S., Min, J.L., Walter, K., Timpson, N.J., UK10K Consortium, Dunham, I., Birney, E., et al. (2016). GARFIELD - GWAS Analysis of Regulatory or Functional Information Enrichment with LD correction. BioRxiv. doi: https://doi.org/10.1101/085738.

26. Zheng, J., Erzurumluoglu, A.M., Elsworth, B.L., Kemp, J.P., Howe, L., Haycock, P.C., Hemani, G., Tansey, K., Laurin, C., Early Genetics and Lifecourse Epidemiology (EAGLE) Eczema Consortium, et al. (2017). LD Hub: a centralized database and web interface to perform LD score regression that maximizes the potential of summary level GWAS data for SNP heritability and genetic correlation analysis. Bioinformatics *33*, 272–279.

27. Bulik-Sullivan, B.K., Loh, P.-R., Finucane, H.K., Ripke, S., Yang, J., Schizophrenia Working Group of the Psychiatric Genomics Consortium, Patterson, N., Daly, M.J., Price, A.L., and Neale, B.M. (2015). LD Score regression distinguishes confounding from polygenicity in genome-wide association studies. Nat. Genet. *47*, 291–295.

28. Bulik-Sullivan, B., Finucane, H.K., Anttila, V., Gusev, A., Day, F.R., Loh, P.-R., ReproGen Consortium, Psychiatric Genomics Consortium, Genetic Consortium for Anorexia Nervosa of the Wellcome Trust Case Control Consortium 3, Duncan, L., et al. (2015). An atlas of genetic correlations across human diseases and traits. Nat. Genet. *47*, 1236–1241.

29. Selvin, E., Burnett, A.L., and Platz, E.A. (2007). Prevalence and risk factors for erectile dysfunction in the US. Am. J. Med. *120*, 151–157.

30. Yavorska, O.O., and Burgess, S. (2017). MendelianRandomization: an R package for performing Mendelian randomization analyses using summarized data. Int. J. Epidemiol. *46*, 1734–1739.

31. McKinney, W., and Others (2010). Data structures for statistical computing in python. In Proceedings of the 9th Python in Science Conference, (Austin, TX), pp. 51–56.

32. Cock, P.J.A., Antao, T., Chang, J.T., Chapman, B.A., Cox, C.J., Dalke, A., Friedberg, I., Hamelryck, T., Kauff, F., Wilczynski, B., et al. (2009). Biopython: freely available Python tools for computational molecular biology and bioinformatics. Bioinformatics *25*, 1422–1423.

33. Arnold, M., Raffler, J., Pfeufer, A., Suhre, K., and Kastenmüller, G. (2015). SNiPA: an interactive, genetic variant-centered annotation browser. Bioinformatics *31*, 1334–1336.

34. Hemani, G., Zheng, J., Elsworth, B., Wade, K.H., Haberland, V., Baird, D., Laurin, C., Burgess, S., Bowden, J., Langdon, R., et al. (2018). The MR-Base platform supports systematic causal inference across the human phenome. Elife 2018;7:e34408.
