# Supplementary material for: GWAS Identifies Risk Locus for Erectile Dysfunction and Implicates Hypothalamic Neurobiology and Diabetes in Etiology
Source: Am J Hum Genet. 2018 Dec 21;104(1):157–63. doi: 10.1016/j.ajhg.2018.11.004 (PMC6323625; doi:10.1016/j.ajhg.2018.11.004)
Supplement: Document S2. Article plus Supplemental Data [file mmc3.pdf]

GWAS Identifies Risk Locus for Erectile Dysfunction and Implicates Hypothalamic Neurobiology and Diabetes in Etiology

Jonas Bovijn,[1,2,14,*] Leigh Jackson,[3,14] Jenny Censin,[1,2,14] Chia-Yen Chen,[4,5,14] Triin Laisk,[6,7,14] Samantha Laber,[1,2,14] Teresa Ferreira,[1] Sara L. Pulit,[1,8,9] Craig A. Glastonbury,[1] Jordan W. Smoller,[5] Jamie W. Harrison,[10] Katherine S. Ruth,[10] Robin N. Beaumont,[10] Samuel E. Jones,[10] Jessica Tyrrell,[10] Andrew R. Wood,[10] Michael N. Weedon,[10,14] Reedik Mägi,[6,14] Benjamin Neale,[4,5,14] Cecilia M. Lindgren,[1,2,8,14] Anna Murray,[10,14,*] and Michael V. Holmes[11,12,13,14]

Erectile dysfunction (ED) is a common condition affecting more than 20% of men over 60 years, yet little is known about its genetic architecture. We performed a genome-wide association study of ED in 6,175 case subjects among 223,805 European men and identified one locus at 6q16.3 (lead variant rs57989773, OR 1.20 per C-allele; $p = 5.71 \times 10^{-14}$), located between *MCHR2* and *SIM1*. *In silico* analysis suggests *SIM1* to confer ED risk through hypothalamic dysregulation. Mendelian randomization provides evidence that genetic risk of type 2 diabetes mellitus is a cause of ED (OR 1.11 per 1-log unit higher risk of type 2 diabetes). These findings provide insights into the biological underpinnings and the causes of ED and may help prioritize the development of future therapies for this common disorder.

Erectile dysfunction (ED) is the inability to develop or maintain a penile erection adequate for sexual intercourse.[1] ED has an age-dependent prevalence, with 20%–40% of men aged 60–69 years affected.[1] The genetic architecture of ED remains poorly understood, owing in part to a paucity of well-powered genetic association studies. Discovery of such genetic associations can be valuable for elucidating the etiology of ED and can provide genetic support for potential new therapies.

We conducted a genome-wide association study (GWAS) in the population-based UK Biobank (UKBB) and the Estonian Genome Center of the University of Tartu (EGCUT) cohorts and hospital-recruited Partners HealthCare Biobank (PHB) cohort. Subjects in UKBB were of self-reported white ethnicity, with subjects in EGCUT and PHB of European ancestry, as per principal components analyses (Supplemental Material and Methods).

ED was defined as self-reported or physician-reported ED using ICD10 codes N48.4 and F52.2, or use of oral ED medication (sildenafil/Viagra, tadalafil/Cialis, or vardenafil/Levitra), or a history of surgical intervention for ED (using OPCS-4 codes L97.1 and N32.6) (Supplemental Material and Methods). The prevalence of ED in the cohorts was 1.53% (3,050/199,352) in UKBB, 7.04% (1,182/16,787) in EGCUT, and 25.35% (1,943/7,666) in PHB (Table S1). Demographic characteristics of the subjects in each cohort are shown in Table S2. The reasons for the different prevalence rates in the three cohorts may include a higher median cohort age for men in PHB (65 years, compared to 59 years in UKBB and 42 years in EGCUT; Table S2), "healthy volunteer" selection bias in UKBB,[2] a lack of primary care data availability in UKBB, and intercultural differences, including "social desirability" bias.[3,4] Importantly, we note that the assessment of exposure-outcome relationships remains valid, despite the prevalence likely not being representative of the general population prevalence.

GWASs in UKBB revealed a single genome-wide significant ($p < 5 \times 10^{-8}$) locus at 6q16.3 (lead variant rs57989773, EAF_{UKBB} [C-allele] = 0.24; OR 1.23; $p = 3.0 \times 10^{-11}$). Meta-analysis with estimates from PHB (OR 1.20; $p = 9.84 \times 10^{-5}$) and EGCUT (OR 1.08; $p = 0.16$) yielded a pooled meta-analysis OR 1.20; $p = 5.71 \times 10^{-14}$ (heterogeneity p value = 0.17; Figures 1A–1C). Meta-analysis of all variants yielded no further genome-wide loci. Meta-analysis of our results with previously suggested ED-associated variants also did not result in any further significant loci (Supplemental Material and Methods; Table S3), nor did X chromosome analysis in UKBB.

[1]Big Data Institute at the Li Ka Shing Centre for Health Information and Discovery, University of Oxford, Oxford OX3 7LF, UK; [2]Wellcome Centre for Human Genetics, Nuffield Department of Medicine, University of Oxford, Oxford OX3 7BN, UK; [3]Institute of Biomedical and Clinical Science, University of Exeter Medical School, University of Exeter, Exeter EX2 5DW, UK; [4]Analytic and Translational Genetics Unit, Massachusetts General Hospital, Boston, MA 02114, USA; [5]Psychiatric & Neurodevelopmental Genetics Unit, Massachusetts General Hospital, Boston, MA 02114, USA; [6]Estonian Genome Center, Institute of Genomics, University of Tartu, Tartu 51010, Estonia; [7]Department of Obstetrics and Gynecology, Institute of Clinical Medicine, University of Tartu, Tartu 50406, Estonia; [8]Program in Medical and Population Genetics, Broad Institute, Cambridge, MA 02142, USA; [9]Department of Genetics, University Medical Center Utrecht, Utrecht, the Netherlands; [10]Genetics of Complex Traits, University of Exeter Medical School, University of Exeter, Exeter EX2 5DW, UK; [11]National Institute for Health Research Oxford Biomedical Research Centre, Oxford University Hospital, Old Road, Oxford OX3 7LE, UK; [12]Clinical Trial Service Unit & Epidemiological Studies Unit (CTSU), Nuffield Department of Population Health, Big Data Institute Building, Roosevelt Drive, University of Oxford, Oxford OX3 7LF, UK; [13]Medical Research Council Population Health Research Unit at the University of Oxford, Nuffield Department of Population Health, University of Oxford, Oxford, UK
[14]These authors contributed equally to this work
*Correspondence: jbovijn@well.ox.ac.uk (J.B.), a.murray@exeter.ac.uk (A.M.)
https://doi.org/10.1016/j.ajhg.2018.11.004.
© 2018 The Authors. This is an open access article under the CC BY license (http://creativecommons.org/licenses/by/4.0/).

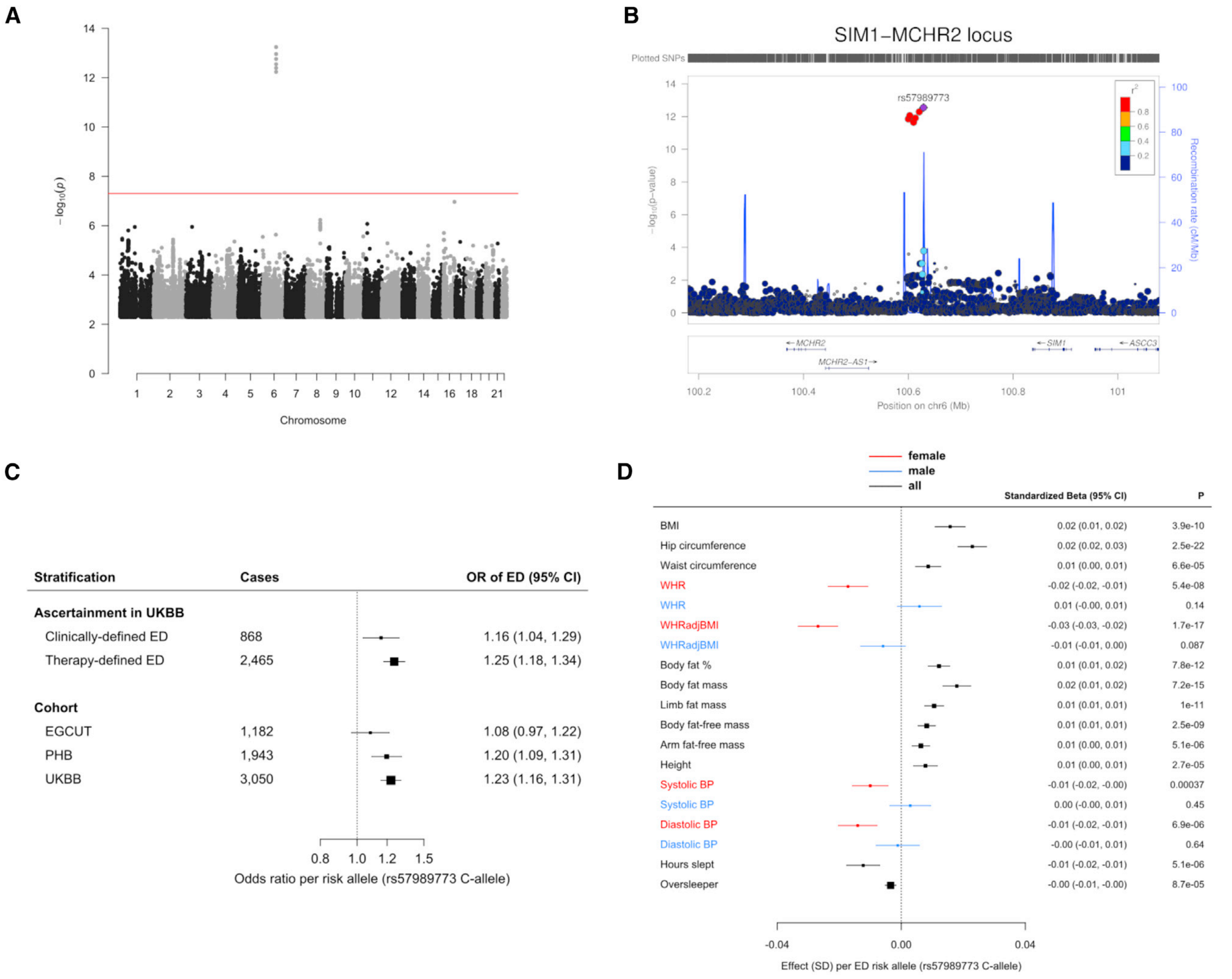

Figure 1. 6q16.3 (Lead Variant rs57989773) Is an Erectile Dysfunction-Associated Locus and Exhibits Pleiotropic Phenotypic Effects
(A) Genome-wide meta-analysis revealed a single genome-wide significant locus for ED at 6q16.3. Only variants with a p value for association of <0.005 are shown. The red line indicates the genome-wide association significance threshold (set at 5×10^{-8}).
(B) Six genome-wide significant variants at 6q16.3 are in high LD.
(C) The association of rs57989773 with ED shows a consistent direction of effect across the three cohorts and across clinically and therapy defined ED in UKBB. Estimates are per C-allele. Boxes represent point estimates of effects. Box sizes are drawn proportional to the precision of the estimates. Lines represent 95% confidence intervals.
(D) PheWAS reveals sex-specific associations of rs57989773 with waist-hip ratio and blood pressure. A PheWAS of 105 predefined traits using the lead ED SNP rs57989773 found associations with 12 phenotypes at $p < 4.8 \times 10^{-4}$ (surpassing the Bonferroni-corrected threshold of 0.05/105; Table S4). All allelic estimates are aligned to the ED risk allele (i.e., C-allele of rs57989773). Due to the nature of the ED phenotype and previously reported sex-specific effects in the *MCHR2-SIM1* locus,[5] sex-specific analyses were performed in significant traits. Diastolic blood pressure (DBP) and systolic blood pressure (SBP) are included here (despite not meeting the Bonferroni-corrected threshold in the original analysis) due to previous reports of effects on blood pressure in individuals with rare, coding variants in *SIM1*. Sexual heterogeneity was found to be present (surpassing a Bonferroni-corrected threshold of 0.05/7 for the number of traits where sex-specific analyses were conducted) for DBP ($p\ value_{heterogeneity} = 6.52 \times 10^{-3}$), SBP ($p\ value_{heterogeneity} = 3.73 \times 10^{-3}$), waist to hip ratio (WHR; $p\ value_{heterogeneity} = 2.39 \times 10^{-6}$), and WHR adjusted for BMI ($p\ value_{heterogeneity} = 1.77 \times 10^{-5}$). This plot shows sex-specific estimates only for traits showing presence of sexual heterogeneity. Continuous traits were standardized prior to analysis to facilitate comparison. Boxes represent point estimates of effects. Box sizes are drawn proportional to the precision of the estimates. Lines represent 95% confidence intervals.

The association of rs57989773 was consistent across clinically and therapy defined ED, as well as across different ED drug classes (Figures 1C and S1). No further genome-wide significant loci were identified for ED when limited to clinically or therapy defined case subjects (2,032 and 4,142 case subjects, respectively).

A PheWAS of 105 predefined traits (Table S4) using the lead ED SNP rs57989773 found associations with 12 phenotypes at a $p\ value < 5 \times 10^{-4}$ (surpassing the Bonferroni-corrected threshold of 0.05/105), including adiposity (nine traits), adult height, and sleep-related traits. Sex-stratified analyses revealed sexual dimorphism for waist-hip ratio (WHR; unadjusted and adjusted for

body mass index) and systolic and diastolic blood pressure (Figure 1D; Table S5).

The lead variant at the 6q16.3 locus, rs57989773, lies in the intergenic region between *MCHR2* and *SIM1*, with *MCHR2* being the closest gene (distances to transcription start sites of 187 kb for *MCHR2* and 284 kb for *SIM1*). Conditional and joint analysis (Supplemental Material and Methods) revealed no secondary, independent signals in the locus. Previous work has implicated the *MCHR2-SIM1* locus in sex-specific associations on age at voice-breaking and menarche.[5] The puberty timing-associated SNP in the *MCHR2-SIM1* region (rs9321659; ~500 kb from rs57989773) was not in LD with our lead variant (r^2 = 0.003, D′ = 0.095) and was not associated with ED (p = 0.32) in our meta-analysis, suggesting that the ED locus represents an independent signal.

To identify the tissue and cell types in which the causal variant(s) for ED may function, we examined chromatin states across 127 cell types[6,7] for the lead variant rs57989773 and its proxies ($r^2 > 0.8$, determined using HaploReg v.4.1) (Supplemental Material and Methods). Enhancer marks in several tissues, including embryonic stem cells, mesenchymal stem cells, and endothelial cells, indicated that the ED-associated interval lies within a regulatory locus (Figure 2A; Table S6).

To predict putative targets and causal transcripts, we assessed domains of long-range three-dimensional chromatin interactions surrounding the ED-associated interval (Figure 2B). Chromosome conformation capture (Hi-C) in human embryonic stem cells[8] showed that *MCHR2* and *SIM1* were in the same topologically associated domain (TAD) as the ED-associated variants, with high contact probabilities (referring to the relative number of times that reads in two 40-kb bins were sequenced together) between the ED-associated interval and *SIM1* (Figures 2B and S2). This observation was further confirmed in endothelial precursor cells,[9] where Capture Hi-C revealed strong connections between the *MCHR2-SIM1* intergenic region and the *SIM1* promoter (Figure 2C), pointing toward *SIM1* as a likely causal gene at this locus.

We next used the VISTA enhancer browser[10] to examine *in vivo* expression data for non-coding elements within the *MCHR2-SIM1* locus. A regulatory human element (hs576), located 30-kb downstream of the ED-associated interval, seems to drive *in vivo* enhancer activity specifically in the midbrain (mesencephalon) and cranial nerve in mouse embryos (Figure 2D). This long-range enhancer close to ED-associated variants recapitulated aspects of *SIM1* expression (Figure 2D), further suggesting that the ED-associated interval belongs to the regulatory landscape of *SIM1*. Taken together these data suggest that the *MCHR2-SIM1* intergenic region harbors a neuronal enhancer and that *SIM1* is functionally connected to the ED-associated region.

Single-minded homolog 1 (*SIM1*) encodes a transcription factor that is highly expressed in hypothalamic neurons.[11] Rare variants in *SIM1* have been linked to a phenotype of severe obesity and autonomic dysfunction,[12,13] including lower blood pressure. A summary of the variant-phenotype associations at the 6q16 locus in human and rodent models is shown in Table S7. Post hoc analysis of association of rs57989773 with autonomic traits showed nominal association with syncope, orthostatic hypotension, and urinary incontinence (Figure S3). The effects on blood pressure and adiposity seen in individuals with rare coding variants in *SIM1* are recapitulated in individuals harboring the common ED-risk variants at the 6q16.3 locus (Figure 1D), suggesting that *SIM1* is the causal gene at the ED-risk locus. SIM1-expressing neurons also play an important role in the central regulation of male sexual behavior as mice that lack the melanocortin receptor 4 (encoded by *MC4R*) specifically in SIM1-expressing neurons show impaired sexual performance on mounting, intromission, and ejaculation.[14] Thus, hypothalamic dysregulation of *SIM1* could present a potential mechanism for the effect of the *MCHR2-SIM1* locus on ED.

An alternative functional mechanism may be explained by proximity of the lead variant (rs57989773) to an arginase 2 processed pseudogene (LOC100129854), a long non-coding RNA (Figure 2A). RPISeq[15] predicts that the pseudogene transcript would interact with the ARG2 protein, with probabilities of 0.70–0.77. Arginine 2 is involved in nitric oxide production and has a previously established role in erectile dysfunction.[16,17] GTEx expression data[18] demonstrated highest mean expression in adipose tissue, with detectable levels in testis, fibroblasts, and brain. Expression was relatively low in all tissues, however, and there was no evidence that any SNPs associated with the top ED signal were eQTLs for the *ARG2* pseudogene or *ARG2* itself.

As a complementary approach, we also used the Data-driven Expression Prioritized Integration for Complex Traits and GWAS Analysis of Regulatory or Functional Information Enrichment with LD correction (DEPICT and GARFIELD, respectively; Supplemental Material and Methods)[19,20] tools to identify gene-set, tissue-type, and functional enrichments. In DEPICT, the top two prioritized gene-sets were "regulation of cellular component size" and "regulation of protein polymerization," whereas the top two associated tissue/cell types were "cartilage" and "mesenchymal stem cells." None of the DEPICT enrichments reached an FDR threshold of 5% (Tables S8–S10). GARFIELD analyses, which assesses enrichment of GWAS signals in regulatory or functional regions in different cell types, also did not yield any statistically significant enrichments, therefore limiting the utility of these approaches in this case.

ED is recognized to be observationally associated with various cardiometabolic traits and lifestyle factors,[21,22] including type 2 diabetes mellitus (T2D), hypertension, and smoking. To further evaluate these associations, we first conducted LD score regression[23,24] to evaluate the genetic correlation of ED with a range of traits. LD score regression identified ED to share the greatest genetic

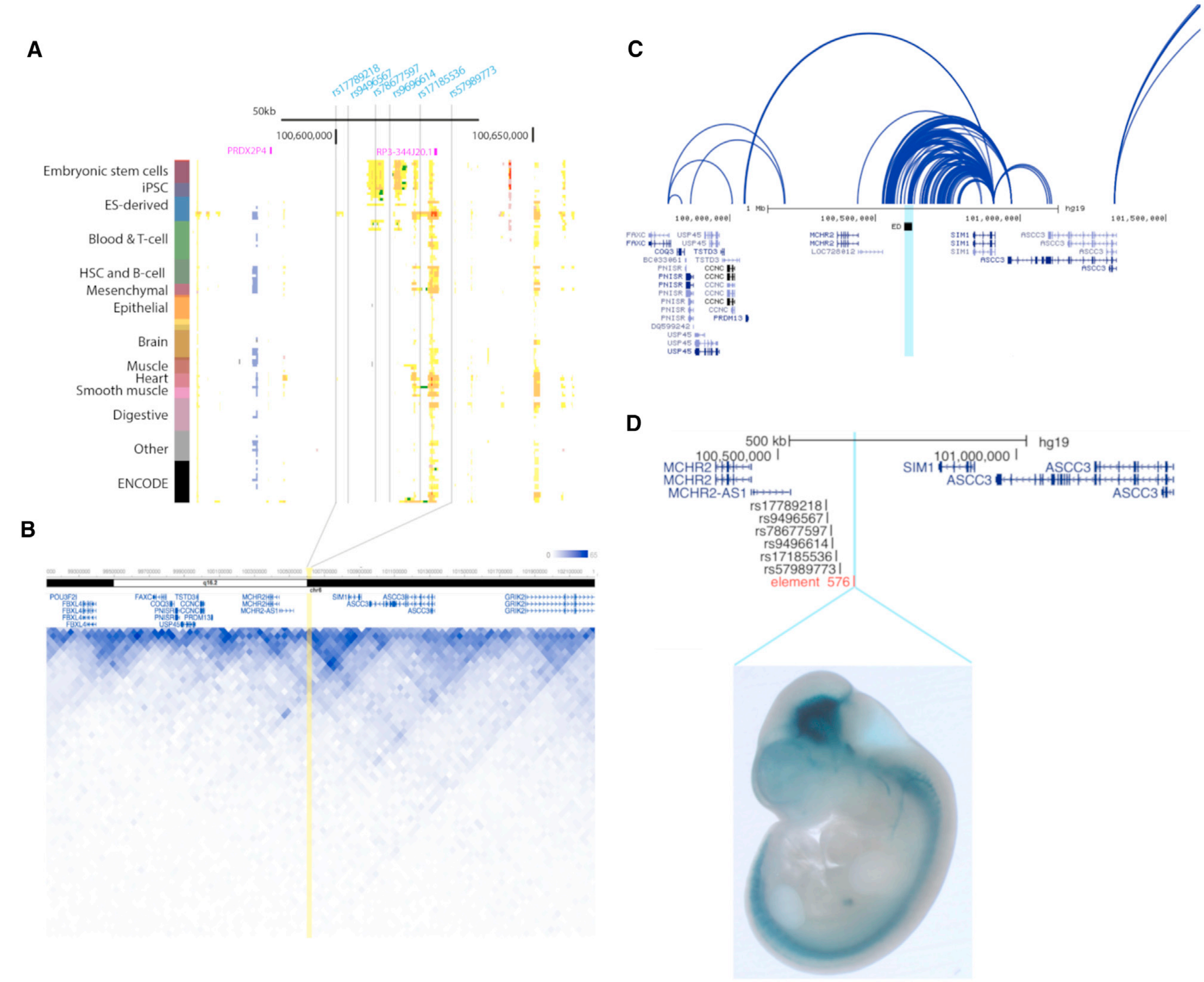

Figure 2. Functional Analysis of 6q16.3 Implicates *SIM1* in ED Pathogenesis

(A) ED-associated signal overlaps regulatory annotations in embryonic stem cells. Chromatin state annotations for the ED-associated region across 127 reference epigenomes (rows) for cell and tissue types profiled by the Roadmap Epigenomics Project.[6,7] Grey vertical lines indicate the position of the ED-associated variant (rs57989773) and its proxies that are in LD $r^2 > 0.8$ determined using HaploReg v4.1[34] (rs17789218, rs9496567, rs78677597, rs9496614, and rs17185536). The lead variant is in proximity to "RP3-344J20.1," an arginase 2 processed pseudogene (LOC100129854).

(B) The ED-associated interval is functionally connected to *SIM1* in embryonic stem cells. The 3D Genome Browser[9] was used to visualize chromosome conformation capture (Hi-C) interactions contact probabilities in human embryonic stem cells,[8] revealing high contact probability between the ED-associated region (highlighted in yellow) and *SIM1* at 40-kb resolution. The heatmap values on a color scale correspond to the number of times that reads in two 40-kb bins were sequences together (blue, stronger interaction; white, little or no interaction).

(C) The *MCHR2-SIM1* intergenic region forms functional connections to the *SIM1* promoter in endothelial progenitors. The 3D Genome Browser[9] was used to visualize Capture Hi-C in endothelial precursors.[35] Light blue vertical line indicates position of the ED-associated interval.

(D) The *MCHR2-SIM1* intergenic region harbors a neuronal enhancer. Top: position of human element hs576 (blue vertical line) and the ED-associated variant rs57989773 and its five proxies in $r^2 > 0.8$ (rs17789218, rs9496567, rs78677597, rs9496614, rs17185536). hs576 is flanked by genes *MCHR2-AS1* and *SIM1*. This panel was generated using the UCSC genome browser.[36] Bottom: expression pattern of human element hs576 in a mouse embryo at e11.5. Expression pattern shows that hs576 drives *in vivo* enhancer activity specifically in mesencephalon (midbrain) and cranial nerve. Embryo image was obtained from the VISTA enhancer browser, with permission from the investigators.[10]

correlation with T2D, limb fat mass, and whole-body fat mass (FDR-adjusted p values < 0.05; Table S11).

Next we performed Mendelian randomization[25] (MR) analyses to evaluate the potential causal role of nine predefined cardiometabolic traits on ED risk (selected based on previous observational evidence linking such traits to ED risk[21]), i.e., T2D, insulin resistance, systolic blood pressure, LDL cholesterol, smoking heaviness, alcohol consumption, body mass index, coronary heart disease, and educational attainment (Tables S12–S15). MR identified

genetic risk to T2D to be causally implicated in ED: each 1-log higher genetic risk of T2D was found to increase risk of ED with an OR of 1.11 (95% CI 1.05–1.17, $p = 3.5 \times 10^{-4}$, which met our *a priori* Bonferroni-corrected significance threshold of 0.0056 [0.05/9]), with insulin resistance likely representing a mediating pathway[26] (OR 1.36 per 1 standard deviation genetically elevated insulin resistance, 95% CI 1.01–1.84, $p = 0.042$). Sensitivity analyses were conducted to evaluate the robustness of the T2D-ED estimate (Figure S5, Table S13), including weighted median analyses (OR 1.12, 95% CI 1.02–1.23, $p = 0.0230$), leave-one-out analysis for all variants (which indicated that no single SNP in the instrument unduly influenced the overall value derived from the summary IVW estimate[27]), and a funnel plot (showing a symmetrical distribution of single-SNP IV estimates around the summary IVW causal estimate). The MR-Egger regression (intercept $p = 0.35$) provided no evidence to support the presence of directional pleiotropy as a potential source of confounding.[28]

We also identified a potential causal effect of systolic blood pressure (SBP), with higher SBP being linked to higher risk of ED (MR-Egger OR 2.34 per 1 standard deviation higher SBP, 95% CI 1.26–4.36, $p = 0.007$, with MR-Egger intercept [$p = 0.007$] suggesting presence of directional pleiotropy). LDL cholesterol (LDL-C) showed minimal evidence of a causal effect (OR 1.07 per 1 standard deviation higher LDL-C, 95% CI 0.98–1.17, $p = 0.113$), and there was limited evidence to support a role for smoking heaviness or alcohol consumption (Table S15). Genetic risk of coronary heart disease (CHD) showed weak effects on risk of ED, suggesting that pathways leading to CHD may be implicated in ED (OR 1.08, 95% CI 1.00–1.17, $p = 0.061$). Further, we identified no causal effects of BMI (using a polygenic score or a single SNP in *FTO*) or education on risk of ED.

Genetic variants may inform drug target validation by serving as a proxy for drug target modulation.[29] ED is most commonly treated using phosphodiesterase 5 (PDE5) inhibitors such as sildenafil. To identify potential phenotypic effects of PDE5 inhibition (e.g., to predict side effects or opportunities for repurposing), we looked for variants in or around *PDE5A*, encoding PDE5, which showed association with the ED phenotype. Of all 4,670 variants within a 1 Mb window of *PDE5A* (chromosome 4:119,915,550–121,050,146 as per GRCh37/hg19), the variant with the strongest association was rs115571325, 26 kb upstream of *PDE5A* (OR_{Meta} 1.25, nominal p value $= 8.46 \times 10^{-4}$; Bonferroni-corrected threshold $[0.05/4,670] = 1.07 \times 10^{-5}$; Figure S6). Given the weak association with ED, we did not evaluate this variant in further detail.

We have gained insight into ED, a common condition with substantial morbidity, by conducting a large-scale GWAS and performing several follow-up analyses. By aggregating data from 3 cohorts, including 6,175 ED-affected case subjects of European ancestry, we identified a locus associated with ED, with several lines of evidence suggesting *SIM1*, highly expressed in the hypothalamus, to be the causal gene at this locus. Our findings provide human genetic evidence in support of the key role of the hypothalamus in regulating male sexual function.[14,30–33]

Mendelian randomization implicated risk of T2D as a causal risk factor for ED with suggestive evidence for insulin resistance and systolic blood pressure, corroborating well-recognized observational associations with these cardiometabolic traits.[22] Further research is needed to explore the extent to which drugs used in the treatment of T2D might be repurposed for the treatment of ED. Lack of evidence for a causal effect of BMI on ED risk in MR analysis (using multiple SNPs across the genome) suggests that the association of the lead SNP (rs57989773) with BMI arises from pleiotropy and that the association of this variant with ED risk is independent of its association with adiposity.

In conclusion, in a large-scale GWAS of more than 6,000 ED-affected case subjects, we provide insights into the biological underpinnings of ED and have elucidated causal effects of various risk factors, including pathways involved in the etiology of T2D. Further large-scale GWASs of ED are needed in order to provide additional clarity on its genetic architecture and etiology and to shed light on potential new therapies.

Data Availability

Full summary statistics of the erectile dysfunction genome-wide meta-analysis are available at the following URL: http://www.geenivaramu.ee/tools/ED_AJHG_Bovijn_et_al_2018.gz and at the LD Hub GWAShare Center at the following URL: http://ldsc.broadinstitute.org/gwashare/.

Supplemental Data

Supplemental Data include 7 figures, 15 tables, and Supplemental Material and Methods and can be found with this article online at https://doi.org/10.1016/j.ajhg.2018.11.004.

Acknowledgments

We thank the UK Biobank (http://www.ukbiobank.ac.uk/; application 11867), Partners HealthCare Biobank (https://biobank.partners.org/), the Estonian Biobank of the Estonian Genome Center of the University of Tartu (https://www.geenivaramu.ee/en), and their participants. EGCUT received funding from the European Commission Horizon 2020 research and innovation programme under grant agreement 692065 (project WIDENLIFE).

S.L. has a Postdoctoral Research Fellowship funded by Novo Nordisk. M.V.H. works in a unit that receives funding from the UK Medical Research Council and is supported by a British Heart Foundation Intermediate Clinical Research Fellowship (FS/18/23/33512). C.M.L. is supported by the Li Ka Shing Foundation, WT-SSI/John Fell funds, Oxford, Widenlife, and NIH (5P50HD028138-27). M.V.H. and C.M.L. are supported by the National Institute for Health Research Oxford Biomedical Research Centre. We gratefully acknowledge all the studies and databases that made GWAS summary data available to LD Hub. These are listed in the Supplemental Data.

Declaration of Interests

M.N.W. has received speaker fees from Ipsen and Merck. B.N. is a member of the scientific advisory board of Deep Genomics and Consultant for Avanir Therapeutics. M.V.H. has collaborated with Boehringer Ingelheim in research, and in accordance with the policy of the Clinical Trial Service Unit and Epidemiological Studies Unit (University of Oxford), did not accept any personal payment.

Received: July 26, 2018
Accepted: November 7, 2018
Published: December 20, 2018

Web Resources

2SampleMR v0.3.4 (R package), https://github.com/MRCIEU/TwoSampleMR
BOLT-LMM v2.3, https://data.broadinstitute.org/alkesgroup/BOLT-LMM/
EPACTS v3.3.0, https://github.com/statgen/EPACTS
EASYQC v9.2, https://www.uni-regensburg.de/medizin/epidemiologie-praeventivmedizin/genetische-epidemiologie/software/
DEPICT v1, https://github.com/perslab/depict
GARFIELD v2, https://www.ebi.ac.uk/birney-srv/GARFIELD/
GCTA v1.26.0, http://cnsgenomics.com/software/gcta/#Overview
HaploReg, http://www.broadinstitute.org/mammals/haploreg/haploreg.php
LD HUB v1.9.0, http://ldsc.broadinstitute.org/
MendelianRandomization v0.2.2 (R package), https://cran.r-project.org/web/packages/MendelianRandomization/index.html
METAL, http://csg.sph.umich.edu/abecasis/metal/
PLINK v1.9, www.cog-genomics.org/plink/1.9/
RPISeq v1.0, http://pridb.gdcb.iastate.edu/RPISeq/references.php
SNPTEST v2.5.2, https://mathgen.stats.ox.ac.uk/genetics_software/snptest/snptest.html#introduction

References

1. Lewis, R.W., Fugl-Meyer, K.S., Corona, G., Hayes, R.D., Laumann, E.O., Moreira, E.D., Jr., Rellini, A.H., and Segraves, T. (2010). Definitions/epidemiology/risk factors for sexual dysfunction. J. Sex. Med. *7*, 1598–1607.
2. Fry, A., Littlejohns, T.J., Sudlow, C., Doherty, N., Adamska, L., Sprosen, T., Collins, R., and Allen, N.E. (2017). Comparison of sociodemographic and health-related characteristics of UK biobank participants with those of the general population. Am. J. Epidemiol. *186*, 1026–1034.
3. Kelly, C.A., Soler-Hampejsek, E., Mensch, B.S., and Hewett, P.C. (2013). Social desirability bias in sexual behavior reporting: evidence from an interview mode experiment in rural Malawi. Int. Perspect. Sex. Reprod. Health *39*, 14–21.
4. Catania, J.A., Gibson, D.R., Chitwood, D.D., and Coates, T.J. (1990). Methodological problems in AIDS behavioral research: influences on measurement error and participation bias in studies of sexual behavior. Psychol. Bull. *108*, 339–362.
5. Day, F.R., Bulik-Sullivan, B., Hinds, D.A., Finucane, H.K., Murabito, J.M., Tung, J.Y., Ong, K.K., and Perry, J.R.B. (2015). Shared genetic aetiology of puberty timing between sexes and with health-related outcomes. Nat. Commun. *6*, 8842.
6. Kundaje, A., Meuleman, W., Ernst, J., Bilenky, M., Yen, A., Heravi-Moussavi, A., Kheradpour, P., Zhang, Z., Wang, J., Ziller, M.J., et al.; Roadmap Epigenomics Consortium (2015). Integrative analysis of 111 reference human epigenomes. Nature *518*, 317–330.
7. Ernst, J., and Kellis, M. (2015). Large-scale imputation of epigenomic datasets for systematic annotation of diverse human tissues. Nat. Biotechnol. *33*, 364–376.
8. Dixon, J.R., Jung, I., Selvaraj, S., Shen, Y., Antosiewicz-Bourget, J.E., Lee, A.Y., Ye, Z., Kim, A., Rajagopal, N., Xie, W., et al. (2015). Chromatin architecture reorganization during stem cell differentiation. Nature *518*, 331–336.
9. Wang, Y., Zhang, B., Zhang, L., An, L., Xu, J., Li, D., Choudhary, M.N.K., Li, Y., Hu, M., Hardison, R., et al. (2017). The 3D Genome Browser: a web-based browser for visualizing 3D genome organization and long-range chromatin interactions. bioRxiv. https://doi.org/10.1101/112268.
10. Visel, A., Minovitsky, S., Dubchak, I., and Pennacchio, L.A. (2007). VISTA Enhancer Browser–a database of tissue-specific human enhancers. Nucleic Acids Res. *35*, D88–D92.
11. Holder, J.L., Jr., Zhang, L., Kublaoui, B.M., DiLeone, R.J., Oz, O.K., Bair, C.H., Lee, Y.-H., and Zinn, A.R. (2004). Sim1 gene dosage modulates the homeostatic feeding response to increased dietary fat in mice. Am. J. Physiol. Endocrinol. Metab. *287*, E105–E113.
12. Ramachandrappa, S., Raimondo, A., Cali, A.M.G., Keogh, J.M., Henning, E., Saeed, S., Thompson, A., Garg, S., Bochukova, E.G., Brage, S., et al. (2013). Rare variants in single-minded 1 (SIM1) are associated with severe obesity. J. Clin. Invest. *123*, 3042–3050.
13. Michaud, J.L., Boucher, F., Melnyk, A., Gauthier, F., Goshu, E., Lévy, E., Mitchell, G.A., Himms-Hagen, J., and Fan, C.M. (2001). Sim1 haploinsufficiency causes hyperphagia, obesity and reduction of the paraventricular nucleus of the hypothalamus. Hum. Mol. Genet. *10*, 1465–1473.
14. Semple, E., and Hill, J.W. (2018). Sim1 neurons are sufficient for MC4R-mediated sexual function in male mice. Endocrinology *159*, 439–449.
15. Muppirala, U.K., Honavar, V.G., and Dobbs, D. (2011). Predicting RNA-protein interactions using only sequence information. BMC Bioinformatics *12*, 489.
16. Cox, J.D., Kim, N.N., Traish, A.M., and Christianson, D.W. (1999). Arginase-boronic acid complex highlights a physiological role in erectile function. Nat. Struct. Biol. *6*, 1043–1047.
17. Lacchini, R., Muniz, J.J., Nobre, Y.T.D.A., Cologna, A.J., Martins, A.C.P., and Tanus-Santos, J.E. (2015). Relationship between Arginase 1 and Arginase 2 levels and genetic polymorphisms with erectile dysfunction. Nitric Oxide *51*, 36–42.
18. GTEx Consortium (2013). The Genotype-Tissue Expression (GTEx) project. Nat. Genet. *45*, 580–585.
19. Pers, T.H., Karjalainen, J.M., Chan, Y., Westra, H.-J., Wood, A.R., Yang, J., Lui, J.C., Vedantam, S., Gustafsson, S., Esko, T., et al.; Genetic Investigation of ANthropometric Traits (GIANT) Consortium (2015). Biological interpretation of genome-wide association studies using predicted gene functions. Nat. Commun. *6*, 5890.
20. Iotchkova, V., Ritchie, G.R.S., Geihs, M., Morganella, S., Min, J.L., Walter, K., Timpson, N.J., UK10K Consortium, Dunham, I., Birney, E., et al. (2016). GARFIELD - GWAS Analysis of Regulatory or Functional Information Enrichment with LD correction. bioRxiv. https://doi.org/10.1101/085738.

21. Selvin, E., Burnett, A.L., and Platz, E.A. (2007). Prevalence and risk factors for erectile dysfunction in the US. Am. J. Med. *120*, 151–157.
22. Yafi, F.A., Jenkins, L., Albersen, M., Corona, G., Isidori, A.M., Goldfarb, S., Maggi, M., Nelson, C.J., Parish, S., Salonia, A., et al. (2016). Erectile dysfunction. Nat. Rev. Dis. Primers *2*, 16003.
23. Bulik-Sullivan, B.K., Loh, P.-R., Finucane, H.K., Ripke, S., Yang, J., Patterson, N., Daly, M.J., Price, A.L., Neale, B.M.; and Schizophrenia Working Group of the Psychiatric Genomics Consortium (2015). LD Score regression distinguishes confounding from polygenicity in genome-wide association studies. Nat. Genet. *47*, 291–295.
24. Bulik-Sullivan, B., Finucane, H.K., Anttila, V., Gusev, A., Day, F.R., Loh, P.-R., Duncan, L., Perry, J.R., Patterson, N., Robinson, E.B., et al.; ReproGen Consortium; Psychiatric Genomics Consortium; and Genetic Consortium for Anorexia Nervosa of the Wellcome Trust Case Control Consortium 3 (2015). An atlas of genetic correlations across human diseases and traits. Nat. Genet. *47*, 1236–1241.
25. Smith, G.D., and Ebrahim, S. (2003). 'Mendelian randomization': can genetic epidemiology contribute to understanding environmental determinants of disease? Int. J. Epidemiol. *32*, 1–22.
26. Wang, Q., Holmes, M.V., Davey Smith, G., and Ala-Korpela, M. (2017). Genetic support for a causal role of insulin resistance on circulating branched-chain amino acids and inflammation. Diabetes Care *40*, 1779–1786.
27. Davies, N.M., Holmes, M.V., and Davey Smith, G. (2018). Reading Mendelian randomisation studies: a guide, glossary, and checklist for clinicians. BMJ *362*, k601.
28. Holmes, M.V., Ala-Korpela, M., and Smith, G.D. (2017). Mendelian randomization in cardiometabolic disease: challenges in evaluating causality. Nat. Rev. Cardiol. *14*, 577–590.
29. Walker, V.M., Davey Smith, G., Davies, N.M., and Martin, R.M. (2017). Mendelian randomization: a novel approach for the prediction of adverse drug events and drug repurposing opportunities. Int. J. Epidemiol. *46*, 2078–2089.
30. Argiolas, A., and Melis, M.R. (2005). Central control of penile erection: role of the paraventricular nucleus of the hypothalamus. Prog. Neurobiol. *76*, 1–21.
31. Argiolas, A., and Melis, M.R. (2013). Neuropeptides and central control of sexual behaviour from the past to the present: a review. Prog. Neurobiol. *108*, 80–107.
32. Courtois, F., Carrier, S., Charvier, K., Guertin, P.A., and Journel, N.M. (2013). The control of male sexual responses. Curr. Pharm. Des. *19*, 4341–4356.
33. Giuliano, F., and Rampin, O. (2004). Neural control of erection. Physiol. Behav. *83*, 189–201.
34. Ward, L.D., and Kellis, M. (2012). HaploReg: a resource for exploring chromatin states, conservation, and regulatory motif alterations within sets of genetically linked variants. Nucleic Acids Res. *40*, D930–D934.
35. Javierre, B.M., Burren, O.S., Wilder, S.P., Kreuzhuber, R., Hill, S.M., Sewitz, S., Cairns, J., Wingett, S.W., Várnai, C., Thiecke, M.J., et al.; BLUEPRINT Consortium (2016). Lineage-specific genome architecture links enhancers and non-coding disease variants to target gene promoters. Cell *167*, 1369–1384.e19.
36. Casper, J., Zweig, A.S., Villarreal, C., Tyner, C., Speir, M.L., Rosenbloom, K.R., Raney, B.J., Lee, C.M., Lee, B.T., Karolchik, D., et al. (2018). The UCSC Genome Browser database: 2018 update. Nucleic Acids Res. *46* (D1), D762–D769.

The American Journal of Human Genetics, Volume *104*

Supplemental Data

GWAS Identifies Risk Locus for Erectile Dysfunction and Implicates Hypothalamic Neurobiology and Diabetes in Etiology

Jonas Bovijn, Leigh Jackson, Jenny Censin, Chia-Yen Chen, Triin Laisk, Samantha Laber, Teresa Ferreira, Sara L. Pulit, Craig A. Glastonbury, Jordan W. Smoller, Jamie W. Harrison, Katherine S. Ruth, Robin N. Beaumont, Samuel E. Jones, Jessica Tyrrell, Andrew R. Wood, Michael N. Weedon, Reedik Mägi, Benjamin Neale, Cecilia M. Lindgren, Anna Murray, and Michael V. Holmes

Figure S1

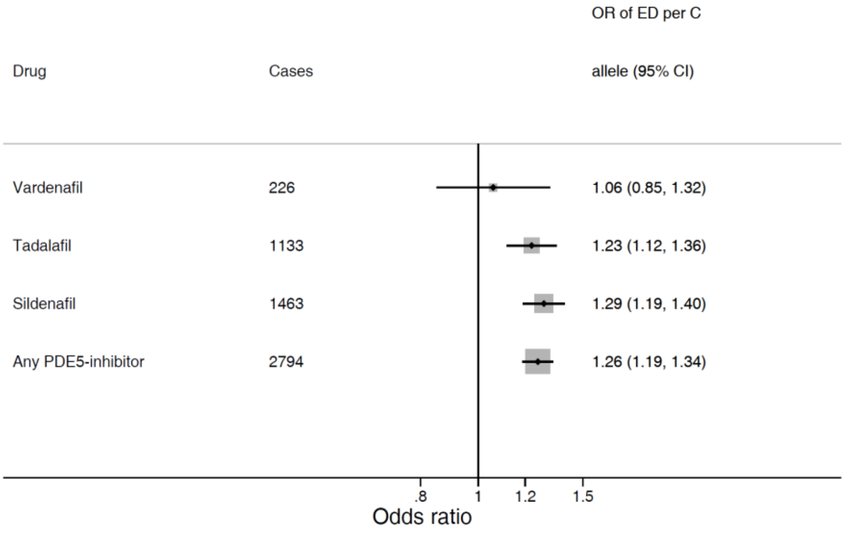

The association of rs57989773 remains consistent across different ED drug classes.

Figure S2

A

B

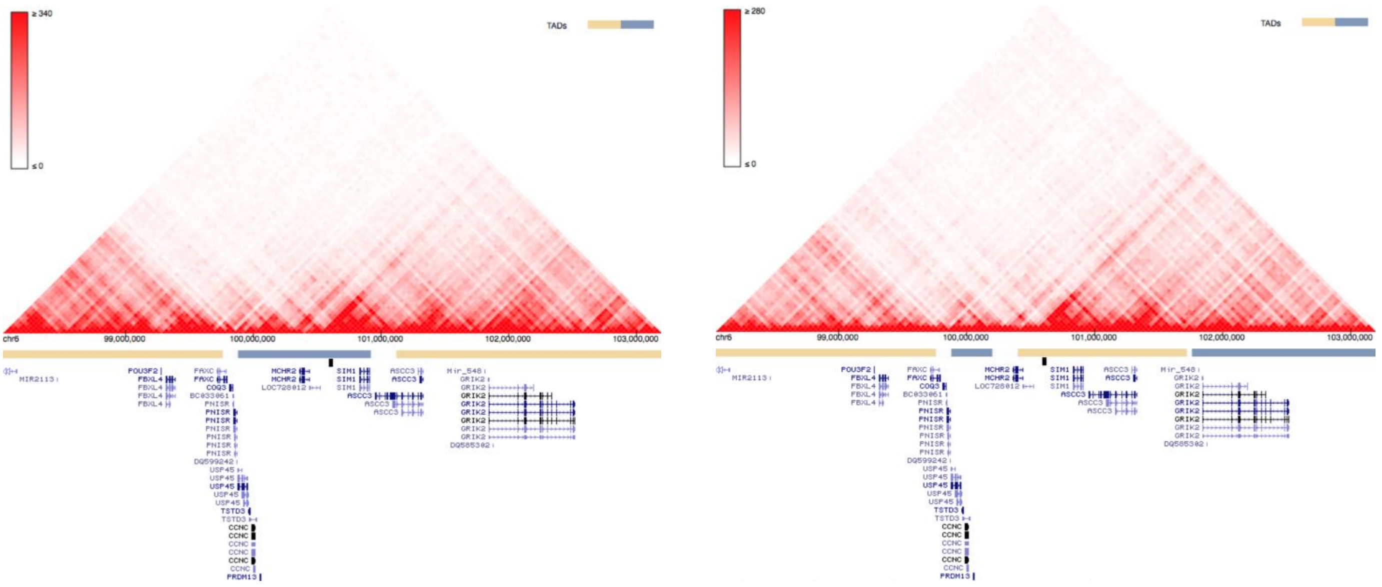

Hi-C interaction maps in several cell types. The 3D Genome Browser[1] was used to visualize the spatial organisation surrounding the ED-associated region. Heatmap shows chromosome conformation capture (Hi-C) interactions contact probabilities in (A) human MES mesendoderm cells[2] at 40-kb resolution; and (B) human mesenchymal stem cells (MSC)[2] at 40-kb resolution. The heat map values on a colour scale correspond to the number of times that reads in two 40-kb bins were sequences together (red - stronger interaction, white - little or no interaction). The second panel indicates the location of the ED-associated region. The third panel shows the UCSC reference genes.

Figure S3

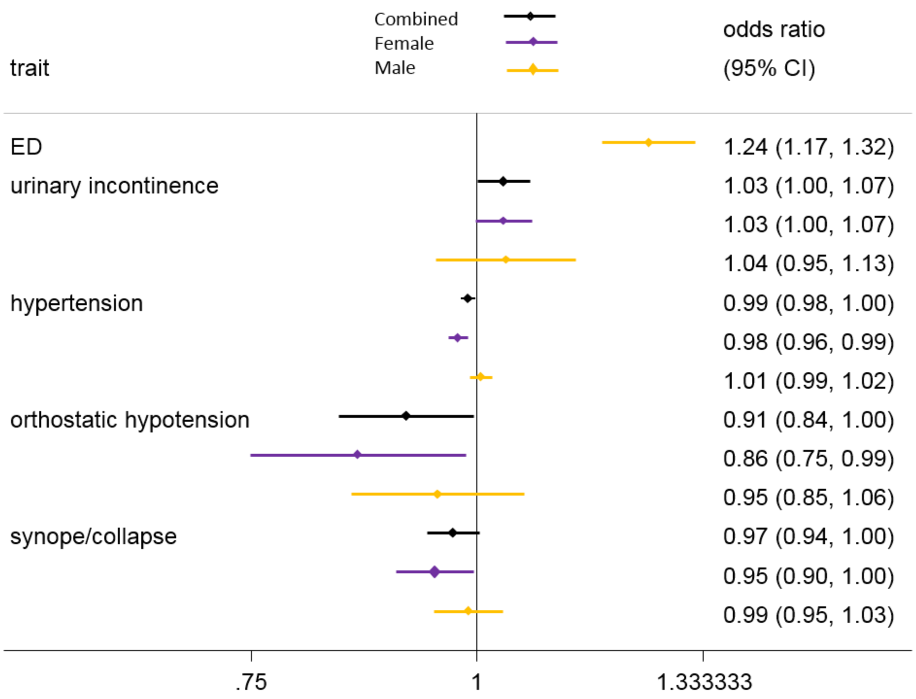

Association of rs57989773 with autonomic phenotypes.

Figure S4

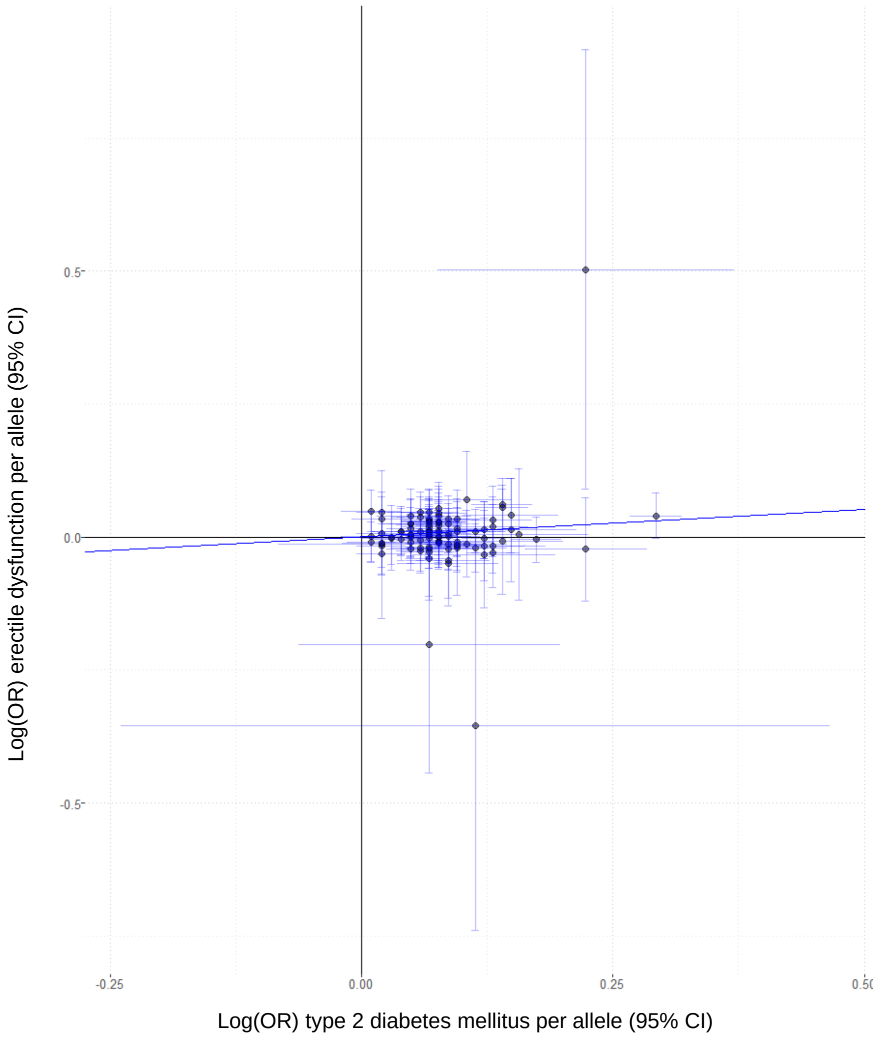

Association of individual SNPs (N=103) with risk for type 2 diabetes mellitus and erectile dysfunction

The blue line represents the inverse variance weighted (IVW) estimate.

Figure S5

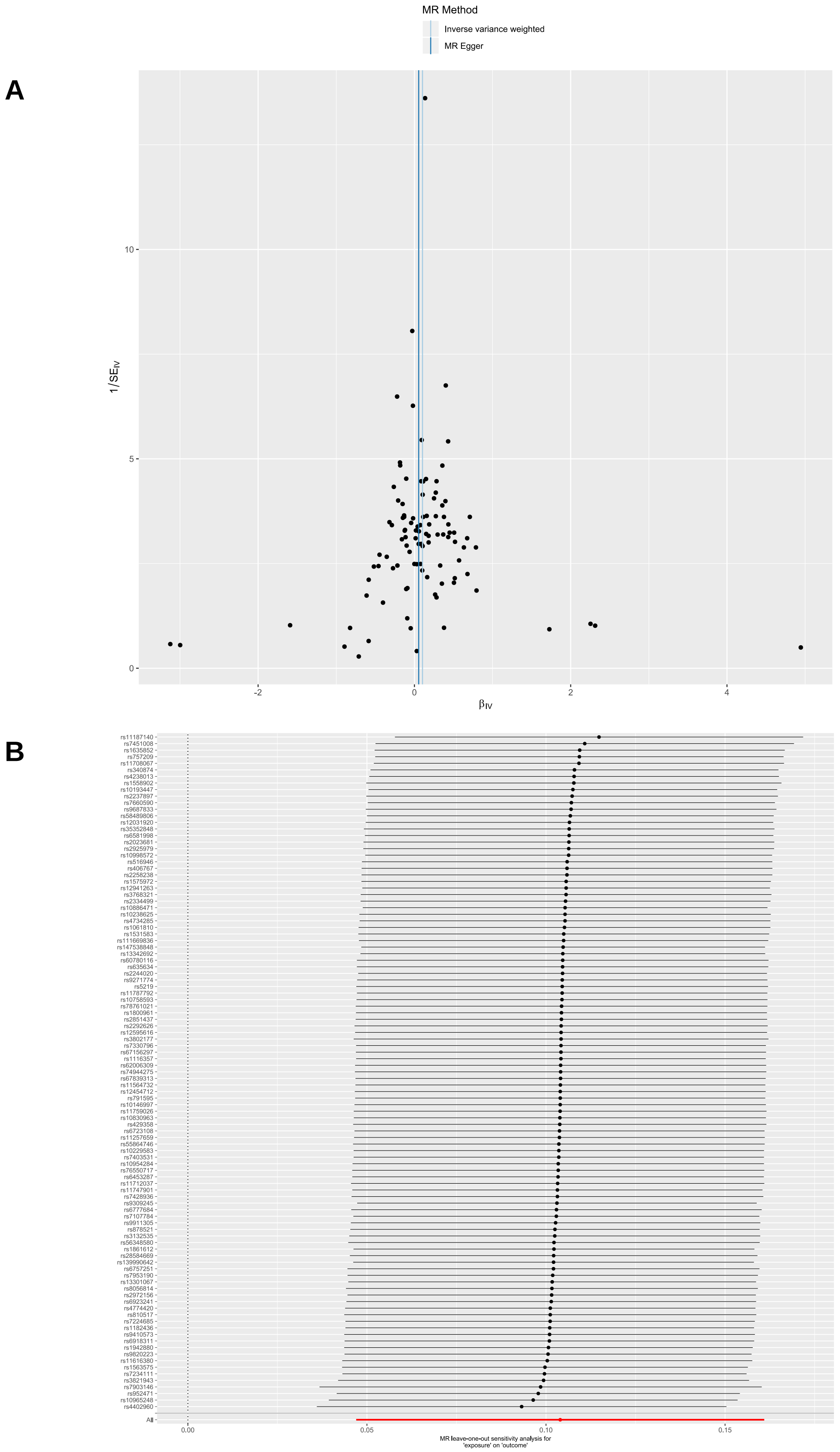

Sensitivity analyses for type 2 diabetes mellitus – erectile dysfunction MR analysis

A. Symmetrical funnel plot of single SNP causal estimates, suggesting absence of directional pleiotropy.

B. Forest plot of leave-one-out analysis (Y-axis indicating which variant was left out for each analysis) indicating that no single SNP in the instrument altered the significance of the IVW estimate (all IVW estimates p-values < 0.0056)

Figure S6

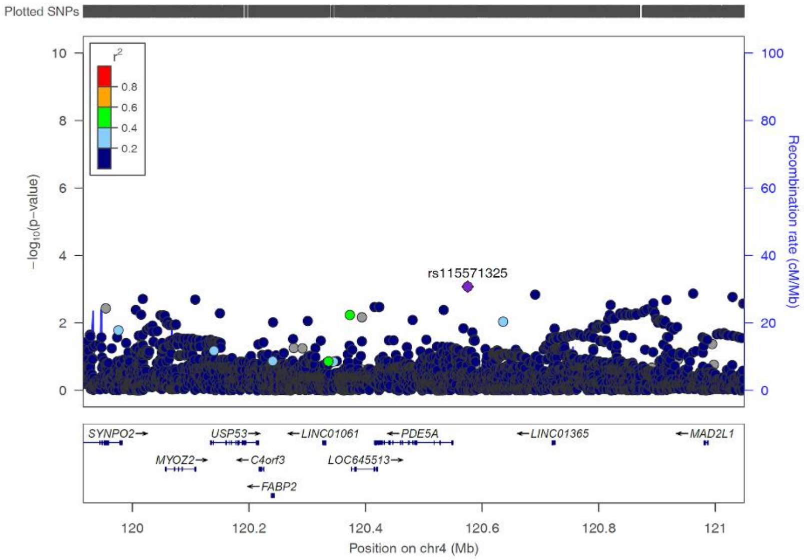

Association of variants in the PDE5A region with ED.

Figure S7

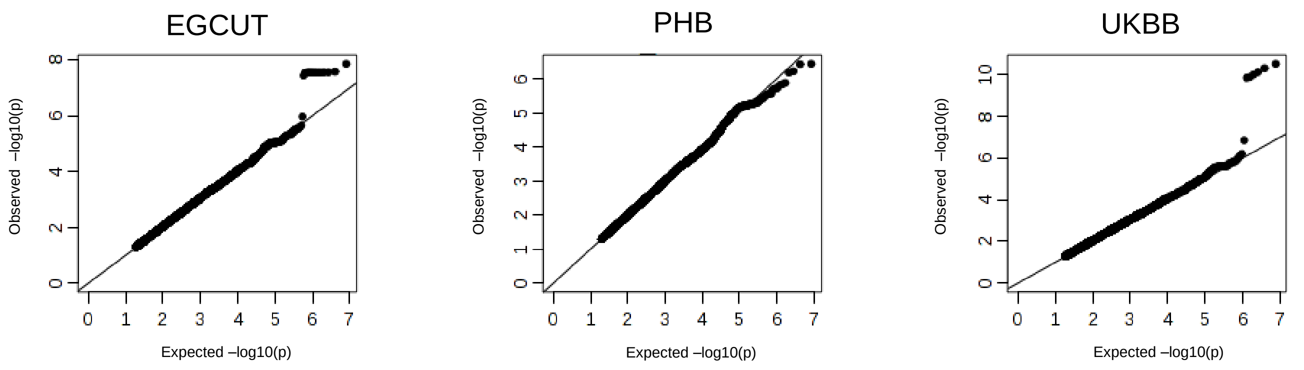

Quantile-quantile (Q-Q) plot of observed versus expected P values in each cohort

Supplemental Material and Methods

STUDY SUBJECTS

Study subjects: Partners HealthCare Biobank

We identified cases of erectile dysfunction (ED) and healthy male controls from the Partners HealthCare Biobank,[3,4] a biorepository of consented patient samples at Partners HealthCare (the parent organization of Massachusetts General Hospital and Brigham and Women's Hospital). All patients who participate in the Partners Biobank are consented for their samples to be linked to their identified clinical information.

The ED cases (Table S1) were identified by querying the Partners Biobank with ICD-10 code N52 (male erectile dysfunction). In addition to ICD-10 code, we also identified ED cases by querying the Partners Biobank with the following drug prescriptions: sildenafil, viagra (25mg, 50mg, and 100mg tablet), tadalafil, cialis (10mg and 20mg tablet), vardenafil, levitra (5mg, 10mg, and 20mg tablet). Given the overlap in indication for phosphodiesterase-5a inhibitors (such as sildenafil and tadalafil), patients with pulmonary hypertension (identified using ICD-10 codes I27.0 and I27.2) were excluded from the identified ED case pool. The controls were males from the Partners Biobank. We also extracted age for ED cases and controls from the Biobank for subsequent analyses. There are 1,943 ED cases and 5,723 male controls with genome-wide genotyped data.

Study subjects: UK Biobank

UK Biobank (UKBB) is a prospective study of more than 500,000 British individuals recruited from 2006 to 2010, aged between 45 and 69.[5] Phenotypic information available includes self-reported medical history (including medication use) as ascertained by verbal interview at enrolment and hospital-derived electronic health record (EHR) data, including International Classification of Disease (ICD-10) diagnosis codes and Office of Population and Censuses

Surveys (OPCS-4) procedure codes.

Individuals in UKBB were defined as having ED (Table S1) on the basis of at least one of the following criteria: Self-reported ED/impotence at time of enrolment; hospitalisation for ICD-10 codes N48.4, F52.2 or N52; hospitalisation for OPCS-4 coded procedures L97.1 or N32.6; or self-reported ED medication (either sildenafil, Viagra, tadalafil, Cialis, vardenafil, Levitra) use. Patients with pulmonary hypertension were excluded from the analyses (identified using ICD-10 codes I27.0 and I27.2, and OPCS-4 codes X821, X822, X823, X824).

Of the 488,377 individuals with available genotype data, we excluded individuals that had: non-white or mixed self-reported ethnicity at any point during follow-up, withdrawn their consent for participation; high sample heterozygosity and missingness; >10 third degree relatives; putative sex chromosome aneuploidy; sex mismatches (genetic vs. self-reported and between assessments); ethnicity mismatches (genetic vs self-reported for White British individuals, and mismatches between assessments) and codes for pulmonary hypertension. After applying these filters, 199,352 male subjects remained, of whom 3,050 met the case-definition criteria for ED, whereas 196,302 subjects served as controls.

Study subjects: Estonian Genome Center of the University of Tartu

The Estonian Genome Center of the University of Tartu (EGCUT) is a population-based biobank with a current cohort size of 51,515 participants.[6] Upon recruitment, the biobank participants filled out a thorough questionnaire, covering lifestyle, diet and clinical diagnoses (described by ICD-10 codes). Data are periodically updated by linking with national health registries. In EGCUT, ED cases (Table S1) were defined using ICD-10 codes F52.2 or N48.4; or data on prescribed drugs (with active compounds tadalafil, sildenafil, vardenafil). Males without any of these diagnosis codes or prescribed drugs were used as controls. The analysis included a total of 1,182 male cases and 15,605 male controls.

GENOTYPING, QC, AND IMPUTATION

Genotyping, QC, and imputation: Partners Healthcare Biobank

DNA samples from the patients in the Partners Biobank were extracted from whole blood. A total of 20,087 samples were genotyped with Illumina Multi-Ethnic Genotyping Array (first batch), Expanded Multi-Ethnic Genotyping Array (second batch), and Multi-Ethnic Global BeadChip (third batch), all of which were designed to capture the global diversity of genetic backgrounds. The number of genotyped variants ranged from 1,416,020 to 1,778,953. We performed QC on each genotyping batch separately as follows: we removed single nucleotide polymorphisms (SNPs) with genotype missing rate > 0.05 before sample-based QC; excluded samples with genotype missing rate > 0.02, absolute value of heterozygosity > 0.2, or failed sex checks; removed SNPs with missing rate > 0.02 after sample-based QC. To merge genotyping batches for imputation and analyses, we performed batch QC by removing SNPs with significant batch association (p-value < 1.0×10^{-6} between different batches). Since the Partners Biobank samples have diverse population backgrounds, we performed Hardy-Weinberg equilibrium test (p-value < 1.0×10^{-6}) for SNP-based QC after extracting samples with European ancestry (see below). We also performed relatedness tests by identifying pairs of samples with $\pi > 0.2$ and excluding one sample from each related sample pair (560 samples excluded). All QC were conducted using PLINK v1.9 and R software.

We extracted samples with European ancestry based on principal component analysis (PCA) with 1000 Genomes Project reference samples. Details of the procedure used to extract European ancestry samples were described previously.[7,8] Briefly, we ran PCA on study samples combined with 1000 Genomes Project reference samples and calculate Euclidean distance (d_{EUR}) for each study sample to the average PC1 and PC2 of the 1000 Genomes Project EUR samples. A total of 16,453 study samples with European ancestry were extracted based on $d_{EUR} < 0.003$. We then performed PCA on the European ancestry samples to obtain PCs for the subsequent analyses.

Genotype imputation was performed on the QCed European ancestry samples with a 2-step pre-phasing/imputation approach. We used Eagle2 for the pre-phasing and minimac3 for imputation, with a reference panel from 1000 Genomes Project phase 3. The final analytic data includes 1,943 ED cases and 5,723 controls of European ancestry with imputed genotype data.

Genotyping, QC, and imputation: UK Biobank

Genotyping, quality control and imputation were performed centrally by UKBB, and details are described elsewhere[9] (see also http://biobank.ctsu.ox.ac.uk/crystal/label.cgi?id=100319). Briefly, genotype data are available for 488,377 individuals, 49,950 of whom were genotyped using the Applied Biosystems™ UK BiLEVE Axiom™ Array by Affymetrix (containing 807,411 markers[10]). The remaining 438,427 individuals were genotyped using the Applied Biosystems™ UK Biobank Axiom™ Array by Affymetrix (containing 825,927 markers). Both of these arrays were specifically designed for use in the UKBB project and share ~95% of marker content. Phasing was done using SHAPEIT3, and imputation was conducted using IMPUTE4. For imputation, the Haplotype Reference Consortium (HRC) panel[11] was used wherever possible, and for SNPs not in that reference panel, a merged UK10K + 1000 Genomes reference panel was used. SNPs were imputed from both panels, but the HRC imputation was preferentially used for SNPs present in both panels. Given known issues with non-HRC imputed SNPs with the current UKBB genotype data release (http://www.ukbiobank.ac.uk/2017/07/important-note-about-imputed-genetics-data/), we included only HRC-imputed SNPs in our dataset.

Genotyping, QC, and imputation: Estonian Genome Center of the University of Tartu

In EGCUT, DNA was extracted from whole blood. Genotyping was carried out using Illumina Human CoreExome, OmniExpress, 370CNV BeadChip and GSA arrays. Genotype array data

was filtered sample-wise by excluding on the basis of call rate (<98%), heterozygosity (>mean±3 SD), genotype and phenotype sex discordance, cryptic relatedness (IBD>20%) and outliers from the European descent based on the MDS plot in comparison with HapMap reference samples. SNP quality filtering included call rate (<99%), MAF (<1%) and extreme deviation from Hardy–Weinberg equilibrium (P-value$<1 \times 10^{-4}$). Imputation was performed on the QCed samples, using SHAPEIT2 for prephasing, the Estonian-specific reference panel[12] and IMPUTE2 with default parameters.

STATISTICAL ANALYSES

Each cohort conducted association analysis using locally supported methods and workflows. Each analysis was adjusted for age, with additional adjustment for principal components (to adjust for population stratification) if logistic regression was used (PHB only). Linear mixed model-based methods (as used in UKBB and EGCUT) do not require the inclusion of principal components to adjust for population stratification[13].

Statistical analyses: Partners Healthcare Biobank

Logistic regression was used to test genome-wide association for ED in PHB, adjusted for 10 PCs and age, using PLINK v1.9[14].

Statistical analyses: UK Biobank

We used BOLT-LMM[13] v2.3 to perform association analyses in UKBB. BOLT-LMM computes statistics for testing association between phenotype and genotypes using a linear mixed model (LMM)[13]. The performance of BOLT-LMM on the UKBB dataset has been previously validated[14], allows for inclusion of related individuals and has been shown to significantly

increase power when compared to traditional linear regression methods[15]. All analyses were adjusted for age.

Statistical analyses: Estonian Genome Center of the University of Tartu

EPACTS v3.3.0 (using option q.emmax) was used to perform association testing in EGCUT, adjusting for age at recruitment and the kinship matrix.

Statistical analyses: Meta-analyses

Prior to meta-analysis, we performed standardized study-level quality control using EASYQC[16]. All three studies (UKBB, PHB, and EGCUT) were subjected to the same QC measures, with inclusion of variants with imputation INFO scores > 0.4 and MAF > 1%. Indels and CNVs were not included in the meta-analysis. Genomic control (GC) correction was applied to each dataset prior to meta-analysis (Pre-correction GC lambda values: UKBB = 1.047; PHB = 1.01; EGCUT = 1.006).

We confirmed that the lead variant at 6q16.3, rs57989773, had acceptable imputation quality in all cohorts (UKBB INFO score: 0.94; PHB INFO score: 0.89; EGCUT INFO score: 0.92). Quantile-quantile (QQ) plots for each cohort are shown in Figure S7.

METAL software[17] was used for performing fixed effect inverse-variance weighted meta-analysis of allelic effect sizes after conversion onto the log-odds scale for the LMM-derived estimates from UKBB and EGCUT. This has been shown to be a valid method for meta-analysis of GWA studies of binary phenotypes using linear mixed models[18].

In addition to the main meta-analysis, we conducted two additional meta-analyses using clinically- or therapy-defined cases respectively. Since only UKBB and PHB had this data available, only these two cohorts were included in these analyses. Identical methodology as described above was followed when performing these meta-analyses.

Statistical analyses: Conditional and joint analysis

We performed conditional and joint analysis of the *MCHR2-SIM1* locus using GCTA[19]. We used individual-level genotype data from UKBB as a reference sample for LD (excluding SNPs with missingness >5%, imputation info score <0.3 and MAF <0.01%, and excluding individuals as for the GWAS analysis in UKBB detailed above). Using relatedness data provided by the UKBB data-release, we reviewed pairwise genetic relationships between individuals and removed one of each pair of individuals with an estimated relatedness of >0.025.

Replication and meta-analysis of previously reported ED-associated SNPs

We performed a literature review for previous ED GWAS and identified three studies[20-22]. We extracted all independent, autosomal SNPs associated with ED with $p \leq 9 \times 10^{-6}$ (i.e. all autosomal SNPs included on the GWAS Catalog database entry for “impotence” or “erectile dysfunction”), yielding 23 SNPs. Summary statistics for these variants were subsequently extracted from our GWAMA results. We then performed effective sample-size weighted Z-score meta-analyses of previously reported summary statistics for 17 variants (3 variants omitted due to MAF < 1% in all three cohorts in our study, 2 variants omitted due to absence of effect size direction in the original report and 1 due to alleles being inconsistent between studies) with summary statistics extracted from our GWAMA (Table S3).

IN-SILICO FUNCTIONAL FOLLOW-UP

UCSC Genome Browser (available at http://genome.ucsc.edu/; December 2013 (GRCh37/hg19) assembly) was used to determine distance of rs57989773 to the transcription start sites of *MCHR2* and *SIM1*.

Phenome-wide Association Scan (PheWAS)

A PheWAS of traits in UKBB was carried out as previously described[23]. Briefly, a range of phenotypes were available in UK Biobank, derived from self-reported questionnaire data, ICD10 diagnoses and baseline measurements at clinic visits as part of the study. We tested the association of the lead ED SNP rs57989773 with a range of phenotypes and traits including: anthropometric, reproductive, cardiovascular, learning/memory and incidence of various diseases (Table S4). Association testing was carried out with inverse normalised phenotypes to account for any skewed distributions, using linear regression models in STATA 13, adjusting for SNP chip type (UKB Axiom or UK BiLEVE), ancestry-principal components 1 to 5 supplied by UK Biobank, test centre and age (or year of birth for age at menarche) with the exception of three traits: hypertension, hypothyroidism and household income. Hypertension and hypothyroidism were tested using logistic regression and household income was tested using ordinal logistic regression. The logistic and ordinal logistic models were adjusted using the same covariates as the linear regression model. Due to the nature of the ED phenotype and previously reported sex-specific effects in the *MCHR2-SIM1* locus, we performed sex-specific analyses on significant traits. Female samples in UKBB were ascertained using self-reported sex, where such reported sex was consistent with genetically determined sex. Samples with discordant self-reported/chromosomal sex, were not included in any analyses. Testing for heterogeneity was performed to assess whether any observed sex-specific effects were significant after accounting for multiple testing.

DEPICT

DEPICT[24] (Data-driven Expression Prioritized Integration for Complex Traits) is a comprehensive pathway analysis tool. We used DEPICT to prioritise likely causal genes at associated loci, and to identify enriched gene sets and tissue and cells types where genes from prioritised loci are highly expressed.

In our study, independent variants were identified in the genome-wide association meta-analysis result using PLINK v.1.9 to clump SNPs at an LD-threshold of r^2=0.1 and a physical distance threshold of 500kb, resulting in 37 independent variants with a p-value threshold of $p < 1 \times 10^{-5}$. SNPs in HLA regions, on sex chromosomes or not present in 1000 Genomes Project were excluded from DEPICT analysis. DEPICT was used to identify tissue and cell type annotations in which genes from associated regions were highly expressed, to identify reconstituted gene sets enriched for genes from associated regions and to prioritize genes within associated regions.

GARFIELD

GARFIELD is a functional enrichment analysis approach described more fully elsewhere[25]. Briefly, GARFIELD is a nonparametric method to assess enrichment of GWAS signals in regulatory or functional regions in different cell-types. The software LD-prunes the GWAS-data before assessing fold enrichment at different p-value thresholds from the GWAS study of interest. To minimize bias, it takes into account LD-structure, gene density, and allele frequencies.

LD Score regression and cross-trait genetic correlation analysis

LD Hub[26] was used to conduct LD Score regression and cross-trait genetic correlation analysis. LD Hub is a centralized database of summary-level GWAS results for >100 diseases/traits from different publicly available resources/consortia and uses a web interface that automates the LD Score regression and cross-trait genetic correlation analysis pipeline.

LD Score regression[27] quantifies the contribution of true polygenic signal and confounding biases, such as cryptic relatedness and population stratification, to inflated distribution of test

statistics in genome-wide association studies, by examining the relationship between test statistics and linkage disequilibrium (LD). The LD Score regression intercept can be used to estimate a more powerful and accurate correction factor than genomic control.

Genetic correlation analysis was conducting using cross-trait LD Score regression[28]. This is a technique estimating genetic correlation that requires only GWAS summary statistics and is not biased by sample overlap.

Mendelian Randomization

Multiple traits have shown association with ED in observational studies, including BMI, educational attainment, hypertension, diabetes, hypercholesterolemia, smoking and cardiovascular disease[29]. We therefore investigated the causal effects of these traits using Mendelian randomization (MR) (Table S12). All MR analyses were performed in R 3.4.3 (R Development Core Team (2008). R: A language and environment for statistical computing. R Foundation for Statistical Computing, Vienna, Austria. http://www.R-project.org) using the *MendelianRandomization* R package[30]. Instrument rsIDs were updated using Python version 3.5.2 (Python Software Foundation. Python Language Reference, version 3.5.2. http://www.python.org), libraries Pandas[31] and Biopython[32], and the NCBI SNP website (Available from: https://www.ncbi.nlm.nih.gov/snp/). Proxies (r^2 >0.8) were identified using SNiPA[33]. Leave-one-out analyses and the funnel plot were created using the *TwoSampleMR* R package[34].

ETHICAL APPROVAL

All analyses in UKBB were performed under UKBB application number 11867. UKBB received ethical approval from the North West Centre for Research Ethics Committee (reference number 11/NW/0382).

Analyses in EGCUT were approved by the Ethics Review Committee of the University of Tartu (243T-12).

The Partners HealthCare Biobank maintains blood and DNA samples from consented patients seen at Partners HealthCare hospitals in the Boston area of Massachusetts. Patients are recruited in the context of clinical care appointments, and also electronically at Partners HealthCare. Biobank subjects provide consent for the use of their samples and data in broad-based research.

ACKNOWLEDGEMENTS

We gratefully acknowledge all the studies and databases that made GWAS summary data available: ADIPOGen (Adiponectin genetics consortium), C4D (Coronary Artery Disease Genetics Consortium), CARDIoGRAM (Coronary ARtery DIsease Genome wide Replication and Meta-analysis), CKDGen (Chronic Kidney Disease Genetics consortium), dbGAP (database of Genotypes and Phenotypes), DIAGRAM (DIAbetes Genetics Replication And Meta-analysis), ENIGMA (Enhancing Neuro Imaging Genetics through Meta Analysis), EAGLE (EArly Genetics & Lifecourse Epidemiology Eczema Consortium, excluding 23andMe), EGG (Early Growth Genetics Consortium), GABRIEL (A Multidisciplinary Study to Identify the Genetic and Environmental Causes of Asthma in the European Community), GCAN (Genetic Consortium for Anorexia Nervosa), GEFOS (GEnetic Factors for OSteoporosis Consortium), GIANT (Genetic Investigation of ANthropometric Traits), GIS (Genetics of Iron Status consortium), GLGC (Global Lipids Genetics Consortium), GPC (Genetics of Personality Consortium), GUGC (Global Urate and Gout consortium), HaemGen (haemotological and platelet traits genetics consortium), HRgene (Heart Rate consortium), IIBDGC (International Inflammatory Bowel Disease Genetics Consortium), ILCCO (International Lung Cancer Consortium), IMSGC (International Multiple Sclerosis Genetic Consortium), MAGIC (Meta-Analyses of Glucose and Insulin-related traits Consortium), MESA (Multi-Ethnic Study of Atherosclerosis), PGC (Psychiatric Genomics Consortium), Project MinE consortium, ReproGen (Reproductive Genetics Consortium), SSGAC (Social Science Genetics Association Consortium) and

TAG (Tobacco and Genetics Consortium), TRICL (Transdisciplinary Research in Cancer of the Lung consortium), UK Biobank. We gratefully acknowledge the contributions of Alkes Price (the systemic lupus erythematosus GWAS and primary biliary cirrhosis GWAS) and Johannes Kettunen (lipids metabolites GWAS).

References

1. Wang, Y., Zhang, B., Zhang, L., An, L., Xu, J., Li, D., Choudhary, M.N.K., Li, Y., Hu, M., Hardison, R., et al. (2017). The 3D Genome Browser: a web-based browser for visualizing 3D genome organization and long-range chromatin interactions. BioRxiv. doi: https://doi.org/10.1101/112268.

2. Dixon, J.R., Jung, I., Selvaraj, S., Shen, Y., Antosiewicz-Bourget, J.E., Lee, A.Y., Ye, Z., Kim, A., Rajagopal, N., Xie, W., et al. (2015). Chromatin architecture reorganization during stem cell differentiation. Nature *518*, 331–336.

3. Smoller, J.W., Karlson, E.W., Green, R.C., Kathiresan, S., MacArthur, D.G., Talkowski, M.E., Murphy, S.N., and Weiss, S.T. (2016). An eMERGE Clinical Center at Partners Personalized Medicine. J Pers Med *6*.

4. Gainer, V.S., Cagan, A., Castro, V.M., Duey, S., Ghosh, B., Goodson, A.P., Goryachev, S., Metta, R., Wang, T.D., Wattanasin, N., et al. (2016). The Biobank Portal for Partners Personalized Medicine: A Query Tool for Working with Consented Biobank Samples, Genotypes, and Phenotypes Using i2b2. J Pers Med *6*.

5. Collins, R. (2012). What makes UK Biobank special? Lancet *379*, 1173–1174.

6. Leitsalu, L., Haller, T., Esko, T., Tammesoo, M.-L., Alavere, H., Snieder, H., Perola, M., Ng, P.C., Mägi, R., Milani, L., et al. (2015). Cohort Profile: Estonian Biobank of the Estonian Genome Center, University of Tartu. Int. J. Epidemiol. *44*, 1137–1147.

7. Stein, M.B., Chen, C.-Y., Ursano, R.J., Cai, T., Gelernter, J., Heeringa, S.G., Jain, S., Jensen, K.P., Maihofer, A.X., Mitchell, C., et al. (2016). Genome-wide Association Studies of Posttraumatic Stress Disorder in 2 Cohorts of US Army Soldiers. JAMA Psychiatry *73*, 695–704.

8. Chen, C.-Y., Pollack, S., Hunter, D.J., Hirschhorn, J.N., Kraft, P., and Price, A.L. (2013). Improved ancestry inference using weights from external reference panels. Bioinformatics *29*, 1399–1406.

9. Bycroft, C., Freeman, C., Petkova, D., Band, G., Elliott, L.T., Sharp, K., Motyer, A., Vukcevic, D., Delaneau, O., O'Connell, J., et al. (2017). Genome-wide genetic data on ~500,000 UK Biobank participants. BioRxiv. doi: https://doi.org/10.1101/166298.

10. Wain, L.V., Shrine, N., Miller, S., Jackson, V.E., Ntalla, I., Soler Artigas, M., Billington, C.K., Kheirallah, A.K., Allen, R., Cook, J.P., et al. (2015). Novel insights into the genetics of smoking behaviour, lung function, and chronic obstructive pulmonary disease (UK BiLEVE): a genetic association study in UK Biobank. Lancet Respir Med *3*, 769–781.

11. McCarthy, S., Das, S., Kretzschmar, W., Delaneau, O., Wood, A.R., Teumer, A., Kang, H.M., Fuchsberger, C., Danecek, P., Sharp, K., et al. (2016). A reference panel of 64,976 haplotypes for genotype imputation. Nat. Genet. *48*, 1279–1283.

12. Mitt, M., Kals, M., Pärn, K., Gabriel, S.B., Lander, E.S., Palotie, A., Ripatti, S., Morris, A.P., Metspalu, A., Esko, T., et al. (2017). Improved imputation accuracy of rare and low-frequency variants using population-specific high-coverage WGS-based imputation reference panel. Eur. J. Hum. Genet. *25*, 869–876.

13. Chang, C.C., Chow, C.C., Tellier, L.C., Vattikuti, S., Purcell, S.M., and Lee, J.J. (2015). Second-generation PLINK: rising to the challenge of larger and richer datasets. Gigascience *4*, 7.

14. Loh, P.-R., Tucker, G., Bulik-Sullivan, B.K., Vilhjálmsson, B.J., Finucane, H.K., Salem, R.M., Chasman, D.I., Ridker, P.M., Neale, B.M., Berger, B., et al. (2015). Efficient Bayesian mixed-model analysis increases association power in large cohorts. Nat. Genet. *47*, 284–290.

15. Loh, P.-R., Kichaev, G., Gazal, S., Schoech, A.P., and Price, A.L. (2018). Mixed model association for biobank-scale data sets. BioRxiv. doi: https://doi.org/10.1101/194944

16. Winkler, T.W., Day, F.R., Croteau-Chonka, D.C., Wood, A.R., Locke, A.E., Mägi, R., Ferreira, T., Fall, T., Graff, M., Justice, A.E., et al. (2014). Quality control and conduct of genome-wide association meta-analyses. Nat. Protoc. *9*, 1192–1212.

17. Willer, C.J., Li, Y., and Abecasis, G.R. (2010). METAL: fast and efficient meta-analysis of genomewide association scans. Bioinformatics *26*, 2190–2191.

18. Cook, J.P., Mahajan, A., and Morris, A.P. (2017). Guidance for the utility of linear models in meta-analysis of genetic association studies of binary phenotypes. Eur. J. Hum. Genet. *25*, 240–245.

19. Yang, J., Ferreira, T., Morris, A.P., Medland, S.E., Genetic Investigation of ANthropometric Traits (GIANT) Consortium, DIAbetes Genetics Replication And Meta-analysis (DIAGRAM) Consortium, Madden, P.A.F., Heath, A.C., Martin, N.G., Montgomery, G.W., et al. (2012). Conditional and joint multiple-SNP analysis of GWAS summary statistics identifies additional variants influencing complex traits. Nat. Genet. *44*, 369–375.

20. Hotaling, J.M., Waggott, D.R., Goldberg, J., Jarvik, G., Paterson, A.D., Cleary, P.A., Lachin, J., Sarma, A., Wessells, H., and DCCT/EDIC Research Group (2012). Pilot genome-wide association search identifies potential loci for risk of erectile dysfunction in type 1 diabetes using the DCCT/EDIC study cohort. J. Urol. *188*, 514–520.

21. Kerns, S.L., Stock, R., Stone, N., Buckstein, M., Shao, Y., Campbell, C., Rath, L., De Ruysscher, D., Lammering, G., Hixson, R., et al. (2013). A 2-stage genome-wide association study to identify single nucleotide polymorphisms associated with development of erectile dysfunction following radiation therapy for prostate cancer. Int. J. Radiat. Oncol. Biol. Phys. *85*, e21–e28.

22. Kerns, S.L., Ostrer, H., Stock, R., Li, W., Moore, J., Pearlman, A., Campbell, C., Shao, Y., Stone, N., Kusnetz, L., et al. (2010). Genome-wide association study to identify single nucleotide polymorphisms (SNPs) associated with the development of erectile dysfunction in African-American men after radiotherapy for prostate cancer. Int. J. Radiat. Oncol. Biol. Phys. *78*, 1292–1300.

23. Tuke, M.A., Ruth, K.S., Wood, A.R., Beaumont, R.N., Tyrrell, J., Jones, S.E., Yaghootkar, H., Turner, C.L.S., Donohoe, M.E., Brooke, A.M., et al. (2017). Phenotypes associated with female X chromosome aneuploidy in UK Biobank: an unselected, adult, population-based cohort. BioRxiv. doi: https://doi.org/10.1101/177659.

24. Pers, T.H., Karjalainen, J.M., Chan, Y., Westra, H.-J., Wood, A.R., Yang, J., Lui, J.C., Vedantam, S., Gustafsson, S., Esko, T., et al. (2015). Biological interpretation of genome-wide association studies using predicted gene functions. Nat. Commun. *6*, 5890.

25. Iotchkova, V., Ritchie, G.R.S., Geihs, M., Morganella, S., Min, J.L., Walter, K., Timpson, N.J., UK10K Consortium, Dunham, I., Birney, E., et al. (2016). GARFIELD - GWAS Analysis of Regulatory or Functional Information Enrichment with LD correction. BioRxiv. doi: https://doi.org/10.1101/085738.

26. Zheng, J., Erzurumluoglu, A.M., Elsworth, B.L., Kemp, J.P., Howe, L., Haycock, P.C., Hemani, G., Tansey, K., Laurin, C., Early Genetics and Lifecourse Epidemiology (EAGLE) Eczema Consortium, et al. (2017). LD Hub: a centralized database and web interface to perform LD score regression that maximizes the potential of summary level GWAS data for SNP heritability and genetic correlation analysis. Bioinformatics *33*, 272–279.

27. Bulik-Sullivan, B.K., Loh, P.-R., Finucane, H.K., Ripke, S., Yang, J., Schizophrenia Working Group of the Psychiatric Genomics Consortium, Patterson, N., Daly, M.J., Price, A.L., and Neale, B.M. (2015). LD Score regression distinguishes confounding from polygenicity in genome-wide association studies. Nat. Genet. *47*, 291–295.

28. Bulik-Sullivan, B., Finucane, H.K., Anttila, V., Gusev, A., Day, F.R., Loh, P.-R., ReproGen Consortium, Psychiatric Genomics Consortium, Genetic Consortium for Anorexia Nervosa of the Wellcome Trust Case Control Consortium 3, Duncan, L., et al. (2015). An atlas of genetic correlations across human diseases and traits. Nat. Genet. *47*, 1236–1241.

29. Selvin, E., Burnett, A.L., and Platz, E.A. (2007). Prevalence and risk factors for erectile dysfunction in the US. Am. J. Med. *120*, 151–157.

30. Yavorska, O.O., and Burgess, S. (2017). MendelianRandomization: an R package for performing Mendelian randomization analyses using summarized data. Int. J. Epidemiol. *46*, 1734–1739.

31. McKinney, W., and Others (2010). Data structures for statistical computing in python. In Proceedings of the 9th Python in Science Conference, (Austin, TX), pp. 51–56.

32. Cock, P.J.A., Antao, T., Chang, J.T., Chapman, B.A., Cox, C.J., Dalke, A., Friedberg, I., Hamelryck, T., Kauff, F., Wilczynski, B., et al. (2009). Biopython: freely available Python tools for computational molecular biology and bioinformatics. Bioinformatics *25*, 1422–1423.

33. Arnold, M., Raffler, J., Pfeufer, A., Suhre, K., and Kastenmüller, G. (2015). SNiPA: an interactive, genetic variant-centered annotation browser. Bioinformatics *31*, 1334–1336.

34. Hemani, G., Zheng, J., Elsworth, B., Wade, K.H., Haberland, V., Baird, D., Laurin, C., Burgess, S., Bowden, J., Langdon, R., et al. (2018). The MR-Base platform supports systematic causal inference across the human phenome. Elife 2018;7:e34408.
